# Artificial Intelligence Detection and Segmentation Models: A Systematic Review and Meta-Analysis of Brain Tumors in Magnetic Resonance Imaging

**DOI:** 10.1016/j.mcpdig.2024.01.002

**Published:** 2024-02-04

**Authors:** Ting-Wei Wang, Yu-Chieh Shiao, Jia-Sheng Hong, Wei-Kai Lee, Ming-Sheng Hsu, Hao-Min Cheng, Huai-Che Yang, Cheng-Chia Lee, Hung-Chuan Pan, Weir Chiang You, Jiing-Feng Lirng, Wan-Yuo Guo, Yu-Te Wu

**Affiliations:** a Institute of Biophotonics, National Yang-Ming Chiao Tung University, Beitou, Taipei, Taiwan; b School of Medicine, College of Medicine, National Yang-Ming Chiao Tung University, Taipei, Taiwan; c Center for Evidence-based Medicine, Taipei Veterans General Hospital, Taipei, Taiwan; d Division of Cardiology, Department of Internal Medicine, Taipei Veterans General Hospital, Taipei, Taiwan; e Institute of Public Health and Community Medicine Research Center, National Yang-Ming University, Taipei, Taiwan; f Department of Medical Education, Taipei Veterans General Hospital, Taipei, Taiwan; g Department of Neurosurgery, Neurological Institute, Taipei Veterans General Hospital, Taipei, Taiwan; h Department of Neurosurgery, Taichung Veterans General Hospital, Taichung, Taiwan; i Department of Medical Research, Taichung Veterans General Hospital, Taichung, Taiwan; j Department of Radiation Oncology, Taichung Veterans General Hospital, Taichung, Taiwan; k Department of Radiology, Taipei Veterans General Hospital, Taipei, Taiwan; l National Yang-Ming Chiao Tung University, Brain Research Center, Taiwan; m National Yang-Ming Chiao Tung University, College Medical Device Innovation and Translation Center, Taiwan

## Abstract

**Objective:**

To thoroughly analyze factors affecting the generalization ability of deep learning algorithms on brain tumor detection and segmentation models.

**Patients and Methods:**

We searched PubMed, Embase, Web of Science, Cochrane Library, and IEEE from inception to July 25, 2023, and 19 studies with 12,000 patients were identified. The criteria required studies to use magnetic resonance imaging (MRI) for brain tumor detection and segmentation, offer clear performance metrics, and use external validation data sets. The study focused on outcomes such as sensitivity and Dice score. Study quality was assessed using QUADAS-2 and CLAIM tools. The meta-analysis evaluated varying algorithms and their performance across different validation data sets.

**Results:**

MRI hardware as the manufacturer may contribute to data set diversity, impacting AI model generalizability. The study found that the best algorithms had a pooled lesion-wise Dice score of 84%, with pooled sensitivities of 87% (patient-wise) and 86% (lesion-wise). Post-2022 methodologies highlighted evolving artificial intelligence techniques. Performance differences were evident among tumor types, likely due to size disparities. 3D models outperformed their 2D and ensemble counterparts in detection. Although specific preprocessing techniques improved segmentation outcomes, some hindered detection.

**Conclusion:**

The study underscores the potential of deep learning in improving brain tumor diagnostics and treatment planning. We also identify the need for further research, including developing a comprehensive diversity index, expanded meta-analyses, and using generative adversarial networks for data diversification, paving the way for AI-driven advancements in oncological patient care.

**Trial Registration:**

PROPERO (CRD42023459108).

Artificial Intelligence (AI) has transitioned from the realms of science fiction into a cornerstone of modern scientific exploration.^1^ Tracing its roots to academic discourse from the mid-20th century, AI’s imprint is evident across sectors, especially in health care, redefining the landscape of brain tumor diagnosis.^2^^,^^3^ Given the intricacies associated with brain tumors and their profound clinical consequences, traditional diagnostic methods, although invaluable, often grapple with the inherent complexities of these conditions. Herein lies the allure of AI: offering diagnostic precision at a speed and efficiency that often surpass human endeavor.^4^^,^^5^

The ascent of AI has been paralleled by the emergence of open-source data sets in the recent decade. With diverse imaging methods and detailed patient data, these data reservoirs have become instrumental for training AI systems.^6^^,^^7^ This democratization of data has catalyzed the integration of AI into conventional brain tumor diagnostic protocols.^8^^,^^9^ However, the very boon of these data abundance also presents challenges, primarily stemming from data set disparities. Factors such as differing imaging standards, equipment variations, and patient diversity can subtly, yet significantly, shape the training and efficacy of AI algorithms.^10^^,^^11^ Recognizing and navigating these disparities is pivotal to harnessing AI’s potential reliability in clinical contexts.^12^ Reflecting on our previous research,^13^ it was evident that the model’s generalization capability was closely tied to data distribution. We hypothesized that although a substantial volume of data could boost performance, the data’s diversity might influence a model’s generalization process equally, suggesting a unique interplay between data set volume and diversity in determining AI efficacy.

As regulatory bodies, including the US Food and Drug Administration, increasingly recognize and endorse AI’s role in health care, it underscores the need to rigorously assess its efficacy in brain tumor diagnostics.^14^ Questions about AI’s parity with human experts, the fidelity of models trained across diverse data sets, and their seamless clinical integration become paramount.^15^ This systematic review and meta-analysis aspires to delve into these inquiries, presenting an overview of the performance of AI models for segmenting and detecting brain tumors in magnetic resonance imaging (MRI) scans and its comparison with health care experts. Drawing from the depth of existing data, our goal is to envision a future where AI seamlessly integrates as an indispensable facet of brain tumor diagnostics.^16^

## Material and Methods

### General Guidelines

This study strictly followed the guidelines by the Preferred Reporting Items for Systematic Reviews and Meta-Analyses extension for Diagnostic Test Accuracy Studies (PRISMA-DTA).^17^ To guarantee compliance with these directives. Details of the checklists associated with PRISMA-DTA for abstracts and the primary PRISMA-DTA can be found in Supplemental Tables 1 and 2 (available online at https://www.mcpdigitalhealth.org/). In addition, this research was registered with PROSPERO, with the registration number CRD42023459108. Given that our study focused on systematic review and meta-analysis, there was no necessity for ethical review board approval or obtaining informed consent from the participants.

### Search Strategy and Selection Criteria

Two independent investigators (T.-W.W. and Y.-C.S.) conducted a comprehensive literature search, including multiple databases such as PubMed, Embase, Web of Science, IEEE, and Cochrane Central, with the details listed in Supplemental Table 3 (available online at https://www.mcpdigitalhealth.org/). This search included records from the initiation of each database up to July 25, 2023. Both investigators systematically evaluated the identified titles and abstracts for relevance. They adopted a collaborative consensus approach, supplemented by reference lists from earlier review articles^4^^,^^6^^,^^18^, ^19^, ^20^, ^21^, ^22^ and additional manual searches. In scenarios where reaching a mutual agreement posed challenges, mediation was sought from the third and senior investigators (M.-S.H. and H.-M.C.), respectively. Inclusion criteria were as follows: studies focusing on pretreatment adult brain tumors using MRI combined with machine or deep learning (DL) techniques, encompassing data from a minimum of 50 patients, offering external validation without retraining with external validation data, yielding both pertinent and quantifiable outcomes, and being full-text articles in English. Exclusionary criteria initially included non-English publications, articles deemed nonrelevant such as reviews or abstracts, studies on nonbrain tumors or pediatric subjects, those using imaging techniques other than MRI or specifically black blood imaging, retracted articles, studies lacking substantial data or external validation, and outcomes not fit for meta-analysis. After the preliminary screening, materials from conferences and supplements were also excluded.

### Data Extraction and Management

Two investigators (T.-W.W. and Y.-C.S.) conducted the data extraction process and focused on essential metrics. Then, we navigated through study attributes, addressing data set criteria, their sources—whether hospitals, research institutes, or public databases—and the standards that informed model assessments and the approaches used to establish ground truths. We analyzed patient demographics, detailing participant counts, data set splits for training and validation/testing, age distributions, and gender demographics, giving special attention to female participation. The performance of models across diverse geographical or temporal split data sets was also evaluated. We assessed 2 distinct methods for data set splitting for models’ validation: a temporal split and a geographical split. The temporal split involved using data sets from a specific period (eg, 2018–2020) for model training and data sets from a different, subsequent period (eg, 2021) for validation. The geographical split model was validated using data from another center or an open-source data set. The temporal and geographical splits were considered forms of external validation, consistent with the methodology outlined in a previous study.^15^

The study evaluated various MRI parameters, including details on slice thickness, scanning modalities, and sequences, including T1-weighted and T2-weighted scans. We assessed the technical caliber of MRI devices by looking into their Tesla ratings, manufacturers, and specific models. Venturing into machine learning/DL methodologies, we spotlighted vital parameters. This involved determining the size of data sets, pinpointing the MRI scans/images earmarked for training, and delving into the finer points of the algorithms, especially their input dimensions. The review concluded with a detailed examination of performance metrics. This involved assessing sensitivities for both patients and lesions, cataloging instances of false positives, and establishing criteria for true positives in detection tasks. Sensitivity, also known as the actual positive rate, was calculated using the formula

$$\text{Sensitivity}=\frac{\text{True}\;\text{Positive}}{\text{True}\;\text{Positive}+\text{False}\;\text{Negative}}$$

Furthermore, the review meticulously explored metrics such as Dice scores, particularly for segmentation tasks. The Dice similarity coefficient, often referred to as Dice score, measures the spatial overlap accuracy of 2 sample sets and is computed as follows:

$$\text{Dice}\;\text{Similarity}\;\text{Coefficient}\;\;\;\;\;\;\;=\frac{2\;\left|\text{Predicted}\;\text{set}\cap \text{True}\;\text{set}\right|}{\left|\text{Predicted}\;\text{set}\right|+\left|\text{True}\;\text{set}\right|}$$

In studies that reported outcomes for varying proportions of tumor segmentation (eg, enhancing tumor, T2 abnormality, and necrotic core), the Dice score about contrast-enhanced lesions was explicitly chosen for analysis.

### Methodologic Quality Appraisal

The quality of the selected studies was rigorously assessed using the Checklist for Artificial Intelligence in Medical Imaging (CLAIM) alongside the Quality Assessment of Diagnostic Accuracy Studies-2 (QUADAS-2).^23^^,^^24^ For the QUADAS-2 assessment, 2 independent investigators (T.-W.W. and Y.-C.S.) took the lead, with any disagreements resolved through mediation by the third and senior investigators (M.-S.H. and Y.-T.W.). Similarly, the CLAIM evaluation (T.-W.W. and J.-S.H.) undertook the primary appraisal, and in instances of a discrepancy, the third and senior investigators (W.-K.L. and Y.-T.W.) were consulted to ensure a consensus-based, comprehensive evaluation.

### Statistical Analysis

For the segmentation task, we conducted a meta-analysis on studies offering Dice scores from out-of-sample external validations, considering both geographically and temporally validation data. We focused on the highest-scoring algorithm if multiple Dice scores were presented in a study due to varying algorithms. We also analyzed results from internal validation data sets separately. Given the varied study populations, we used a random-effects model for analysis,^25^ visualized through forest plots. Our subgroup analyses considered factors such as publication year, clinical parameters (eg, validation methods and tumor types), and technical aspects of model construction, such as input sequences, dimensionality, specific algorithms, and image preprocessing steps. Meta-regression sought correlations between Dice scores and elements such as training data size or MRI machine variations. Furthermore, 2 additional meta-analyses reviewed results across all validation data sets. We used traditional 2-level random-effects and 3-level meta-analysis models clustered by data set to understand technical factors affecting model performance. Then, we explored sources of heterogeneity using the ANOVA test and examined the impact of the moderator.

For the detection task, we conducted 4 meta-analyses. The initial pair, with and without the internal validation set, focused on the best-reported algorithms to examine model generalizability, emphasizing patient-wise and lesion-wise sensitivity. Next, 2 meta-analyses considered all algorithms across every validation data set, spotlighting technical model construction. Finally, we assessed health care professionals alone and in tandem with AI models to gauge AI’s clinical impact. To understand study heterogeneity, we used the *Q* test, considering a *P* value of <.05 as significant. Heterogeneity was categorized using *I*^2^ statistics: not essential (0%-25%), low (26%-50%), moderate (51%-75%), and high (76%-100%).^26^ To address potential publication bias, Egger method was applied to discern funnel plot asymmetry.^27^ All statistical tasks were performed in STATA, except the exploratory analysis of the 3-level random effect model with metafor package in R. A *P* value of <.05 (2-tailed) was deemed statistically significant.

## Results

### Study Identification and Selection

**Figure 1:**
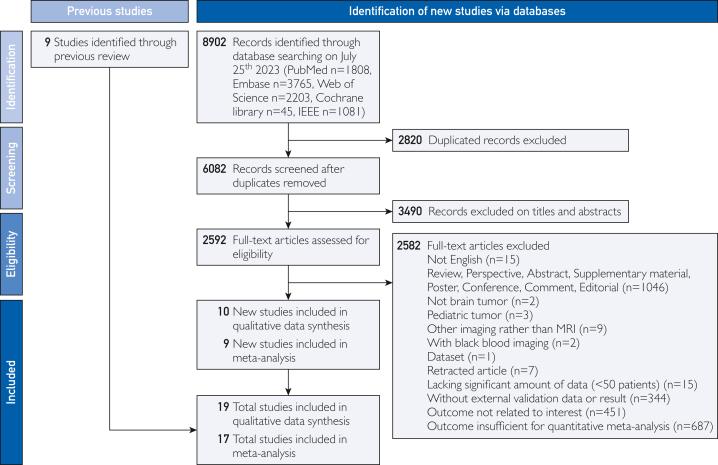
PRISMA flowchart of included studies.

The systematic methodology used for our literature review is outlined within the PRISMA flowchart (Figure 1). Spanning diverse databases, our initial tally constituted 8902 studies, with PubMed (1808), EMBASE (3765), Web of Science (2203), the Cochrane Library (45), and IEEE (1,081) being the primary sources. After removing 2820 duplicates, 6082 articles were identified for further consideration based on their abstracts and titles, using EndNote for screening. A deeper dive into these highlighted 2490 articles that needed more relevance or the necessary depth, prompting their exclusion. Of the 2592 full-text articles examined, a significant 2582 were deemed unfit for inclusion owing to varied factors—ranging from their nature (eg, reviews, editorials, and non-English content) to their relevance (eg, nonbrain tumors, pediatric tumors, and other imaging modalities) and quality (eg, lack of external validation data and insufficient outcomes for meta-analysis). After filtration, a list of 19 pivotal studies emerged,^28^, ^29^, ^30^, ^31^, ^32^, ^33^, ^34^, ^35^, ^36^, ^37^, ^38^, ^39^, ^40^, ^41^, ^42^, ^43^, ^44^, ^45^, ^46^ setting the foundation for our analysis.

### Essential Characteristics of Included Studies

Table 1 presents 19 studies conducted between 2017 and 2023; patient cohorts ranged from 14,046 to 166,534 across Asia, Europe, and North America, with 12,000 patients. Although most studies were retrospective, Yin et al^29^ used a prospective design. Studies varied in structure, encompassing single and multicenter settings, focusing primarily on metastasis, meningioma, and glioma. Data set segmentation into training and testing sets was prevalent, with manual validation consistently used, emphasizing the importance of human intervention in model validation. Lesion size reporting showed disparities; Qu et al^28^ cited 14,542 lesions, in contrast to Sunwoo et al^46^ who reported only 47. The absence of detailed lesion dimensions in studies by Pflüger et al,^30^ Ottesen et al,^31^ and Ma et al^36^ highlight potential characterization gaps. Concerning external validation, 12 studies used geographical validation,^30^, ^31^, ^32^, ^33^, ^34^, ^35^, ^36^^,^^38^^,^^42^, ^43^, ^44^, ^45^, ^46^ three used temporal validation,^39^^,^^41^^,^^46^ and 4 incorporated both.^28^^,^^29^^,^^37^^,^^40^

**Table 1:** Basic Characteristics, Demographics, and External Validation Method of Studies.

Study	Study characteristics			Demographics						External validation	
	Study design	Country	Region	Tumor type	Patients	Series (train/test)	Age, means (SD) or median (range)	Sex female	Lesion size	Geographical validation	Temporal validation
Qu et al,^27^ 2023	Retrospective	China	Asia	Metastasis	1592	1392 (850/492)	I train: 57 (16/NR); I test: 56.6 (10.1/NR); G:17 (10/NR); T1 BM: 58 (15.8/NR); T2 10 mm: 61 (10/NR); 20 mm: 59 (10/NR)	I train: 370 (0.44); I test: 63 (0.42); G: 45 (0.45); T1 BM: 120 (0.53); T2 10 mm:12 (0.35); 20 mm:12 (0.43)	I train: 5; I test 4; G: 5; T1 BM: 6; T2 10 mm: 11; 20 mm: 20.6 (median diameter in mm)	Yes	Yes
Yin et al,^28^ 2022	Prospective	China	Asia	Metastasis	1250	1250 (680/570)	I train BM: 57 (11/NR); I test BM: 57 (10/NR); T BM: 60 (12/NR); G1 BM: 62 (11/NR); G2 BM: 58 (12/NR); G3 BM: 59 (9/NR)	I train BM: 178 (0.43); I test BM: 75 (0.29); T BM: 20 (0.45); G1 BM: 17 (0.45); G2 BM: 19 (0.56); G3 BM: 11 (0.37)	I train BM: 5.5; I test BM: 7.5; T BM: 7.5; G1 BM: 7.9; G2 BM: 6.4; G3 BM: 6.7 (mean diameter in mm; largest cross-sectional dimension on the axial image)	Yes	Yes
Pflüger et al,^29^ 2022	Retrospective	Germany	Europe	Metastasis	338	338 (246/92)	I train: 66 (11/NR); I test: 61 (12/NR); G:58 (12/NR)	I train: 134 (0.55); I test: 29 (0.47); G: 15 (0.5)	I train: 1.23; I test: 1.24; G:1.04 (mean volume in cm^3^); I train: 0.07; I test: 0.05; G:0.08 (median volume in cm^3^)	Yes	No
Ottesen et al,^30^ 2022	Retrospective	Norway	Europe	Metastasis	221	221 (105/116)	I NR (NR/32∼86); G NR (NR/32∼92)	I: 35 (0.6); G: 105 (0.67)	NR	Yes	No
Liang et al,^31^ 2022	Retrospective	America	North America	Metastasis	407	407 (326/81)	I:62 (NR/NR); G:62 (NR/NR)	I:150 (0.46); G:50 (0.62)	I:15.9; G:17.6 (median size in mm)	Yes	No
Kang et al,^32^ 2022	Retrospective	South Korea	Asia	Meningioma	659	659 (459/200)	I train:58 (NR/52-66); I test:62 (NR/55-67); G test:59 (NR/52-65)	I train:74 (0.34); I test:13 (0.13); G test:18 (0.18)	I train: 2.36; I test: 5.06; G:3.05 (median volume in cm^3^)	Yes	No
Chakrabarty et al,^33^ 2022	Retrospective	America	North America	Glioma	1665	1665 (1251/414)	G1:56 (NR/44-46); G2:58 (NR/44-65)	G1:154 (0.40); G2:15 (0.5)	NR	Yes	No
Abayazeed et al,^34^ 2022	Retrospective	America	North America	Glioma	366	366 (300/66)	I train: 52.3 (NR/24-81); I test: 64.5 (NR/14-86); G: 58.6 (NR/32-94)	I train: 223 (0.4); I test 39 (0.56); G: 10 (0.25)	NR	Yes	No
Ma et al,^35^ 2022	Retrospective	China	Asia	Meningioma	651	551 (400/150)	NR	NR	NR	Yes	No
Chen et al,^36^ 2022	Retrospective	China	Asia	Meningioma	609	609 (307/302)	I: 51 (NR/17-79); T: 52.5 (NR/5-86); G: 49 (NR/13-78)	I: 232 (75.57); T:171 (71.85); G:37 (57.81)	I train: 44; I test: 37; G:42 (mean volume in cm^3^)	Yes	Yes
Yi et al,^37^ 2021	Retrospective	Norway	Europe	Metastasis	221	221 (100/121)	NR	NR	NR	Yes	No
Rudie et al,^38^ 2021	Retrospective	America	North America	Metastasis	413	563 (463/100)	61 (12/NR)	238 (57.6)	0.05 (median diameter in cm^3^)	No	Yes
Cho et al,^39^ 2021	Retrospective	South Korea	Asia	Metastasis	194	261 (174/87)	I:62 (12/NR); G:61 (12/NR); T1: 63 (13/NR); T2: 67 (12/NR)	I:80 (0.50); G:16 (0.46); T1:8 (0.40); T2:6 (0.50)	I:6.5; G:7.3; T:6 (median size in mm)	Yes	Yes
Laukamp et al,^40^ 2021	Retrospective	Germany	Europe	Meningioma	126	126 (70/56)	I:58.1 (13.5/24-85); T:59.1 (13.7/33-86)	I:47 (0.67); T:28 (0.5)	I:31.5; T:30.7 (mean volume in cm^3^)	No	Yes
Takahashi et al,^41^ 2021	Retrospective	Japan	Asia	Glioma	544/335	604 (268/336)	G: 60 (NR/86-17)	G: 251 (0.46)	NR	Yes	No
Grøvik et al,^42^ 2021	Retrospective	Norway	Europe	Metastasis	165	165 (100/65)	I:64 (NR/32-92)/G:65 (NR/32-86)	I:71 (0.71); G: 35 (0.54)	NR	Yes	No
Conte et al,^43^ 2021	Retrospective	Italy	Europe	Glioma	277	277 (168/109)	G: 58 (11/NR)	G: 19 (0.41)	NR	Yes	No
Bouget et al,^44^ 2021	Retrospective	Netherlands, Italy, France, America, Austria, Norway	Europe, North America	Glioma	1827	1827 (1534/293)	NR	I: 600 (0.38)	NR	Yes	No
Sunwoo et al,^45^ 2017	Retrospective	South Korea	Asia	Metastasis	140	140 (80/60)	I:60.4 (12/NR); T:63.5 (11.7/NR)-	I:38 (0.475); T:30 (0.5)	I: 5; T:4.5 (median diameter in mm)	No	Yes

BM, brain metastasis; G, geographical validation; I, internal validation; NR, not recorded; T, temporal validation.

### Characteristics of the MRI Machines

The array of imaging techniques depicted in research studies reveals a diverse scanning approach Supplemental Table 5 (available online at https://www.mcpdigitalhealth.org/). Our review found that 9 studies^28^, ^29^, ^30^, ^31^^,^^33^^,^^36^, ^37^, ^38^, ^39^, ^40^ used both 3.0T and 1.5T MRI systems. In addition, 3 studies^28^^,^^31^^,^^39^ exclusively used 1.5T systems, and 1 study^32^ did not specify the Tesla strength. Several studies^30^^,^^31^^,^^38^ used diverse MRI sequences, with 31 melding 2D and 3D techniques, while 28 fixed on 3D imaging alone. A consistent feature observed across the studies was incorporating at least 1 contrast-enhanced sequence. It was observed that contrast-enhanced studies were consistently included, aligning with their critical role in defining contrast-enhancing tumors. A range of scanners was used in some studies, including United Imaging-uMR 588, Siemens-Avanto, and GE-SIGNA Architect, with specific studies opting for different scanners during training and validation phases. Certain studies used other scanners for training and validation phases; for instance, Yin et al^29^ used Siemens MAGNETOM Aera for training and GE Optima MR360 for validation. The image slice thickness varied significantly across studies, ranging from 0.43 to 7.22 mm.

### Characteristics and Performance of DL Algorithms

Most embraced DL methods,^28^, ^29^, ^30^, ^31^, ^32^, ^33^, ^34^, ^35^, ^36^, ^37^, ^38^, ^39^, ^40^, ^41^, ^42^, ^43^, ^44^, ^45^, ^46^ with only 1 using machine learning approach^46^ (Table 2). Data set sizes for training ranged from a minimal 70^41^ to a generous 1534.^45^ Input specifications differed: some studies chose 2D,^28^^,^^33^^,^^36^ others 3D,^29^^,^^30^^,^^32^^,^^35^ and few mixed both.^31^^,^^38^^,^^40^ Imaging sequences spanned T1c,^28^, ^29^, ^30^, ^31^, ^32^, ^33^, ^34^, ^35^, ^36^^,^^39^, ^40^, ^41^, ^42^, ^43^, ^44^, ^45^, ^46^ T1,^30^, ^31^, ^37^, ^38^, ^39^ T2,^35^ FLAIR,^30^, ^31^, ^32^^,^^35^^,^^37^^,^^38^ and more. Regarding algorithms, U-net variants led,^29^, ^30^, ^31^, ^32^^,^^35^^,^^37^^,^^40^ but others such as GHR-convolution neural network (CNN)^28^ and ILD-model^43^ surfaced. Inference times ranged from a quick 4.06 seconds^28^ to a more extensive 264 seconds.^46^ The Dice score, indicating segmentation accuracy, was common, with results ranging from 0.7^39^ to 0.96.^29^ Some reported precision and recall,^28^^,^^33^ whereas a few cited the Harsdorf distance^36^^,^^37^ for spatial accuracy. Patient-wise sensitivity, gauging lesion identification, had metrics from 0.76^31^ to 0.87.^46^ Lesion-wise sensitivity, determining the efficacy of individual lesion detection, varied between 0.65^31^ and 0.96.^29^ Metrics such as true positive and false positive revealed model precision, with false positives from 0.1^31^ to a significant 302.^46^

**Table 2:** Artificial Intelligence Model and Result

Study	Artificial intelligence model							Detection				Segmentation				
	Open code	Training size	Input Dimension	Input sequence	Algorithms name	Algorithms type	Inference time (s)	Patient-wise Sensitivity	Lesion-wise Sensitivity	False positive	True positive definition	Dice	Average method	Precision	Recall	HD
Qu et al,^28^ 2023	NR	850	2D	T1c	GHR-CNN	CNN	4.06	NR	T1: 0.815	T1: 1.65	At least 1 voxel overlapping with ground truth	I: 0.9	Lesion-wise	I: 0.93	I:0.88	NR
Yin et al,^29^ 2022	NR	680	3D	T1c	Multiscale cascaded convolutional network	U-net variation	NR	NR	I: 0.96; T: 0.96; G1: 0.955; G2: 0.889; G3: 0.929	I: 0.39; T: 0.27; G1: 0.36; G2: 0.66; G3:0.69	NR	NR	NR	NR	NR	NR
Pflüger et al,^30^ 2022	NR	246	3D	T1, T1c, FLAIR, T1c-T1	HD-BM	nn-Unet	NR	NR	NR	NR	NR	I:0.78; G:0.79	Lesion-wise	I:0.79; G:0.85	I:0.81; G:0.76	NR
Ottesen et al,^31^ 2022	NR	105	2D/3D Ensemble	T1, T1c, FLAIR, BRAVO	2.5D U-net	U-net	NR	I: 0.76; G: 0.85	I: 0.65; G:0.78	I: 1.7; G: 0.1	Prediction had a 10% or larger overlap	I: 0.85; G: 0.94	Slice-wise^a^	NR	NR	NR
Liang et al,^32^ 2022	NR	326	3D	T1c, FLAIR	KN128_IN	U-net variation	120s	NR	I: 0.86; G: 0.91	I:1.9; G:1.7	One or more Voxels overlap with the ground truth	I:0.73; G: 0.73	Lesion-wise	NR	NR	NR
Kang et al,^33^ 2022	NR	459	2D	T1c	2D nnU-net	nnU-net	NR	NR	NR	NR	NR	I:0.922; G:0.893	Lesion-wise	I:0.933; G:0.905	I:0.917; G:0.915	NR
Chakrabarty et al,^34^ 2022	NR	1251	3D	T1c, T1, T2, FLAIR	3D CNN	U-net variation	NR	NR	NR	NR	NR	I:0.923; G1:0.91; G2: 0.90^b^	Lesion-wise	NR	NR	NR
Abayazeed et al,^35^ 2022	NR	390	3D	T1c, T1, T2, FLAIR	3D U-net	U-net	NR	NR	NR	NR	NR	I:0.89; G:0.82	Lesion-wise	NR	NR	NR
Ma et al,^36^ 2022	NR	400	2D	T1c	DB-HDC with CBAM	CNN	15.076	NR	NR	NR	NR	I1:0.87; I2:0.84; G:0.88^b^	Lesion-wise	I1:0.90; I2:0.85; G:0.88	I1:0.89; I2:0.88; G:0.890	I1:2.52; I2:2.98; G:2.62
Chen et al,^37^ 2022	Yes	307	3D	T1c, T1, T2	Modified attention U-net	U-net variation	NR	NR	NR	NR	NR	G: 0.9^b^	Lesion-wise	G: 0.93	NR	G: 1.99
Yi et al,^38^ 2021	NR	100	2D/3D Ensemble	T1, T1c, FLAIR, BRAVO	2.5D DeepLabv3	CNN	30	NR	I:0.86; G:0.92	NR	Center of mass within 1mm of the expert-annotated lesions	NR	NR	NR	NR	NR
Rudie et al,^39^ 2021	NR	463	3D	T1, T1c, T1c-T1	3D U-net	U-net	45	G: 0.825	G: 0.7	NR	A connected component of the manual segmentation that had any overlap with the predicted segmentation	G: 0.73	Lesion-wise	NR	NR	G: 1.9
Cho et al,^40^ 2021	NR	174	2D/3D Ensemble	T1c	3D U-net + 2D U-net	U-net	NR	NR	G: 0.758; T1: 0.58; T2: 0.80	G: 7.6; T1: 2.5; T2: 2.2	NR	G: 0.66; T1:0.69; T2: 0.82	Lesion-wise	NR	NR	NR
Laukamp et al,^41^ 2021	NR	70	3D	T1c, T1, T2, FLAIR	DeepMedic	CNN	NR	NR	NR	NR	NR	T: 0.91^b^	Lesion-wise	NR	NR	NR
Takahashi et al,^42^ 2021	NR	268	3D	T1c, T1, T2, FLAIR	3D U-net	U-net	NR	NR	NR	NR	NR	G: 0.717	Lesion-wise	G: 0.207	NR	NR
Grøvik et al,^43^ 2021	NR	100	2D/3D Ensemble	T1, T1c, FLAIR, BRAVO	ILD-model	CNN	NR	NR	NR	NR	NR	G: 0.8	Lesion-wise	G: 0.79	G: 0.61	NR
Conte et al,^44^ 2021	Yes	168	3D	T1c, T2, T2 FLAIR	HD-GLIO	U-net variation	NR	NR	NR	NR	NR	I: 0.92; G: 0.86	Lesion-wise	NR	NR	NR
Bouget et al,^45^ 2021	NR	1534	3D	T1c	nnU-net	nnU-net	110	NR	NR	NR	NR	G: 0.84	NR	NR	NR	G: 4.07
Sunwoo et al,^46^ 2017	NR	80	3D	T1c	ML	ANN	264	T: 0.87	NR	T: 302	NR	NR	NR	NR	NR	NR

ANN, artificial neural network; CNN, convolution neural network; DL, deep learning; G, geographical validation; HD, Harsdorf distance; I, internal validation; ML, machine learning; NR, not recorded; T, temporal validation.

a Exclude from quantitative analysis of segmentation performance as calculation Dice score method significantly different from others.

b Select segmentation result of enhancing tumor.

### Quality Assessment

Supplemental Figure 1 (available online at https://www.mcpdigitalhealth.org/) offers a quality assessment overview of the incorporated studies using the QUADAS-2 tool. A detailed breakdown, according to bias risk and applicability concerns, is available in Supplemental Table 9 (available online at https://www.mcpdigitalhealth.org/). Concerning bias risk in patient selection, 7 studies^31^^,^^34^, ^35^, ^36^^,^^38^^,^^39^^,^^43^ exhibited an unclear risk owing to omitting the data set’s interval derivation. Regarding inappropriate exclusions, studies^38^^,^^43^ disregarded nodules based on size criteria. Supplemental Table 10 (available online at https://www.mcpdigitalhealth.org/) gives a thorough quality assessment of the 19 studies using the CLAIM criteria. The studies presented an average CLAIM score of 28.07, with a standard deviation (SD) of 3.51, and scores between 18.00 and 31.00. The mean scores of CLAIM’s subsections were title/abstract at 1.84 (SD = 0.37), introduction consistently at 2.00 (SD = 0.00), and methods at 21. Methods was broken down into study design (1.89, SD = 0.32), data (5.53, SD = 0.84), ground truth (3.26, SD = 1.48), data preparation (2.00, SD = 0.00), model (1.68, SD = 0.67), training (2.00, SD = 0.94), and evaluation (4.63, SD = 0.68). For results, the total was 3.1, subdivided into data (1.21, SD = 0.71) and model performance (1.89, SD = 0.66). The discussion segment scores were 1.89 (SD = 0.32), and the other information section scores were 1.26 (SD = 0.56).

### Efficacy of AI Segmentation of Brain Tumor on MR Images

**Figure 2:**
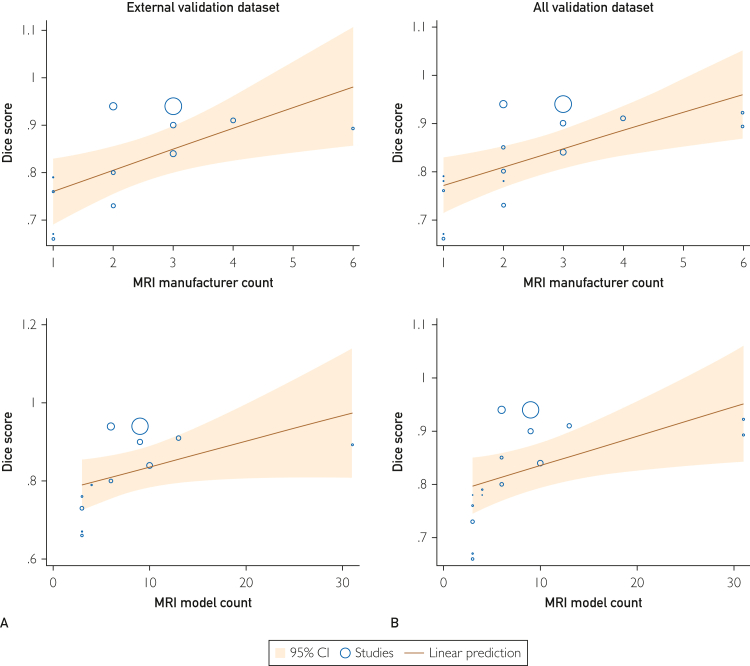
Bubble plot of meta-regression of deep learning algorithms’ lesion-wise Dice score restricted to algorithms reporting the highest accuracy on magnetic resonance imaging (MRI) manufacture count and MRI model count. (A) External validation set: manufacturer count (coefficient: 0.0443; *P*=.01); model count (coefficient: 0.0066; *P*=.065). (B) All validation set: manufacturer count (coefficient: 0.0376; *P*=.003); model count (coefficient: 0.0044; *P*=.023).

When analyzing 15 top algorithms results on external validation data sets for segmentation, Dice scores ranged from 66% to 91%, with an average of 83% (95% CI: 78%-87%). The *Q* test yielded a value of 1002 (*P*<.01), and the Higgins *I*^*2*^ statistic showed a 99.8% variance. Sensitivity analysis confirmed these findings (Supplemental Figure 2A, available online at https://www.mcpdigitalhealth.org/). A more granular subgroup analysis (Supplemental Figure 3A, available online at https://www.mcpdigitalhealth.org/) indicated that certain factors—such as publication year (*P*<.01), tumor subtype (*P*<.01), model input dimension (*P*<.01), N4 bias field correction (*P*<.01), and pixel resampling (*P*=.03)—played pivotal roles in influencing performance. By contrast, factors such as region, validation method, model input sequence, skull stripping, data augmentation, intensity normalization, and image cropping affected less. Meta-regression indicated a correlation between the count of MRI manufacturers (*P*=.01) and MRI model (*P*=.065) with model performance (Figure 2A).

Evaluating the top 20 algorithms results across all validation data sets, the Dice score was 84% on average (95% CI: 81%-88%). The *Q* value was 1262 (*P*<.01), with a Higgins *I*^*2*^ variance of 99.55%. Subgroup analysis found several influential variables on model performance, with pixel resampling’s influence less significant and intensity normalization more (Supplemental Figure 3B, available online at https://www.mcpdigitalhealth.org/). The meta-regression (Figure 2B) confirmed significant correlations between the count of MRI manufacturers (*P*<.01) and MRI models (*P*=.023) with model performance. However, the training data set’s size did not exhibit a significant relationship.

Considering all 58 reported algorithms results, the average Dice score was 83% (95% CI: 81%-85%). The *Q* value was 5257 (*P*<.01), and Higgins *I*^*2*^ showed a variance of 99.61%. Clinical features influencing performance remained consistent in subgroup analysis. Regarding technical variables related to model construction, the model input dimension, algorithm type, N4 bias field correction, data augmentation, and intensity normalization showed significant differences among subgroups (Supplemental Figure S10, available online at https://www.mcpdigitalhealth.org/). Moreover, nnU-net outperformed U-net variations in an algorithmic breakdown, followed by U-net and other CNN architectures.

Using a 3-level meta-analysis model clustered by data sets, the Dice score averaged 82% (95% CI: 79%-85%). The *Q* test (*Q* = 5260, *P*<.01) and Higgins *I*^*2*^ showed a 100% variance. This model showed a significant difference compared with the reduced 2-level model without within data set variance (*P*<.01). However, a borderline significance was observed against the reduced 2-level model without between-data set variance (*P*=.14). Further analysis indicated that the heterogeneity was distributed as 0.41% in level 1, 84.93% in level 2, and 14.66% in level 3. Moderators, such as tumor type, algorithm types, model input dimension, N4 bias field correction, and data augmentation, significantly contributed to this heterogeneity.

### Efficacy of AI Detection of Brain Tumors on MR Images and Comparison With Health Care Expert

In our detection task focused on brain metastasis tumors, we analyzed 17 of the foremost algorithms results applied to all validation data sets. Sensitivity scores ranged between 57% and 96%, and the collective patient-wise sensitivity averaged 87% with a 95% CI of 83%-91% (Figure 12A, available online at https://www.mcpdigitalhealth.org/). The Q test yielded 77.94 (*P*<.01, with Higgins *I*^*2*^ indicating 81% variance. Sensitivity analysis, illustrated in Figure S13A (available online at https://www.mcpdigitalhealth.org/), confirmed these results. Subgroup evaluation shown in Figure S15A (available online at https://www.mcpdigitalhealth.org/) spotlighted variables such as MRI scan dimension (*P*<.01), algorithm type (*P*<.01), pixel resampling (*P*=.01), and intensity normalization (*P*<.01) as pivotal to performance. Meta-regression connected the number of MRI manufacturers (*P*=.017) and MRI model (*P*<.01) to model efficiency (Figures S17A, available online at https://www.mcpdigitalhealth.org/).

**Figure 3:**
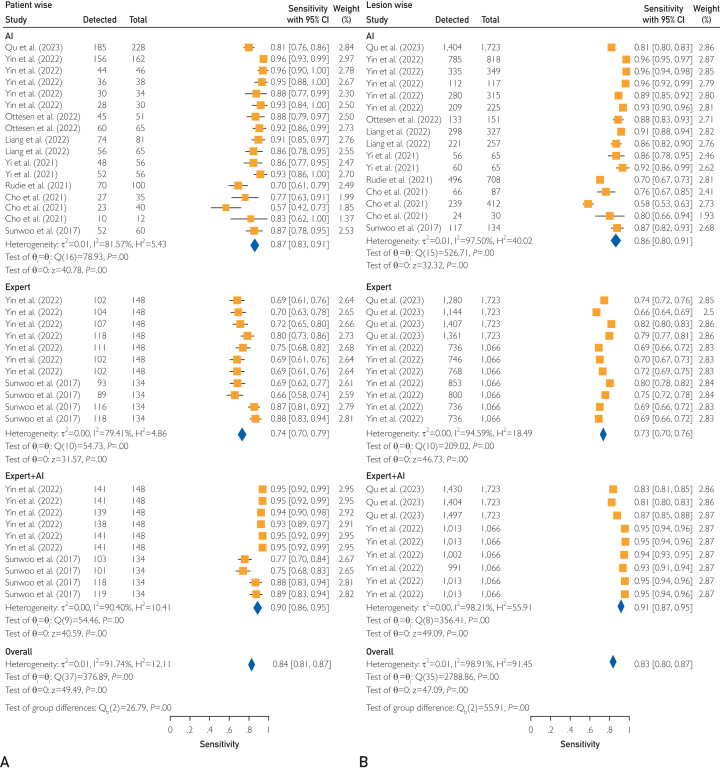
Forest plot of algorithms’ sensitivity restricted to algorithms reporting the highest accuracy on all validation data set compared with (A) health care expert and with (B) health care expert with the aid of algorithm.

Regarding lesion-wise sensitivity, figures ranged from 58% to 96%. The aggregated average was 86%, with a 95% CI of 80%-91% (Figure 12B, available online at https://www.mcpdigitalhealth.org/). Significant variance was evident through the *Q* test (Q = 526.71; *P*<0.01) and Higgins *I*^*2*^ at 97.50%. Sensitivity analysis is shown in Figure S13B (available online at https://www.mcpdigitalhealth.org/). Influencing factors, revealed in Figure S15B (available online at https://www.mcpdigitalhealth.org/), included publication year (*P*=.01), MRI scan dimension (*P*<.01), algorithm type (*P*<.01), pixel resampling (*P*=.01), and intensity normalization (*P*<.01). Meta-regression affirmed a strong correlation between lesion count (*P*<.01) and performance (Figures S16B, available online at https://www.mcpdigitalhealth.org/). Compared with health care experts, AI-assisted experts excelled beyond individual algorithms and solo health care professionals (Figure 3A, B).

### Deep Dive Into the Influence of Analysis Method on Assessing Algorithms Technical Factor Relation to Performance AI Model

The influence of technical factors on algorithm segmentation performance is detailed in Supplemental Table 7 (available online at https://www.mcpdigitalhealth.org/). Although training size showed no significant impact, suggesting larger data sets do not always yield superior results, model input dimensions and algorithm types emerged as critical drivers with consistent significance (*P*<.01 across data sets). Notably, preprocessing methods such as N4 bias field correction and pixel resampling varied significantly based on the analytical method (*P*<.01 in some, nonsignificant in others).

A few pivotal trends emerge from the comprehensive data presented in Supplemental Table 8 (available online at https://www.mcpdigitalhealth.org/) regarding the role of algorithm-related technical factors in moderating detection performance. For patient-wise sensitivity, it is clear that training size consistently showed a lack of significance across all validation sets. Conversely, the lesion number manifested some importance, especially in the external validation set (*P*=.028). Preprocessing steps, such as the N4 bias field correction and data augmentation, are markedly influential in select data sets (both at *P*<.01), revealing their potential role in fine-tuning segmentation outcomes. The model input dimension and algorithm types are pivotal parameters recurring across multiple validation sets. On the contrary, for lesion-wise sensitivity, most of the technical factors, including training size and lesion number, displayed varied levels of significance across different validations. The model input sequence and model input dimension showed significant effects in several data sets (both at *P*<.01), emphasizing their potential role in enhancing lesion detection accuracy.

## Discussion

This study represents the first systematic review and meta-analysis focused on the generalizability of AI models in brain tumor detection and segmentation. In our research, we identified several factors that contribute to the diversity of data sets, which in turn may impact the generalizability of these AI models. After carefully selecting studies with transparent reporting of diagnostic performance and validation of the algorithm with external validation data sets, we found that brain tumor type and MRI hardware, such as manufacturers and companies that design, produce, and sell MRI machines, may contribute to the diversity of data sets. In addition, technical factors in constructing the AI model also significantly impact the final performance of the model.

Our initial assumption posited that the diversity of a data set would influence the generalizability of a model trained on it. This hypothesis found support through a meta-regression, where the MRI manufacturer and MRI model acted as moderators affecting the algorithm’s segmentation Dice score on external data sets. The impact of the MRI manufacturer was significant (*P*=.01), whereas the MRI model was on the cusp of significance (*P*=.065). However, there are limitations to this analysis. We only considered the MRI hardware from the training data and did not account for or match the MRI hardware from the validation data, mainly owing to a lack of detailed information from the studies. Furthermore, the relationship among the MRI manufacturers, the MRI models, and how algorithms interact with the data might be more intricate than a straightforward linear relationship based on the count. For detection tasks, the dimension of MRI scans revealed significant variations in the subgroup sensitivity of the top-performing algorithms on an external validation set. Models using a 2D/3D ensemble approach outperformed those relying on 3D alone. These findings bolster the idea that data set diversity, encompassing MRI hardware and protocols before model development, profoundly impacts a model’s performance on external validation sets. A more varied data set fosters enhanced generalizability.

There was a notable correlation with the publication year in segmentation and detection tasks; studies published post-2022 outperformed earlier publications. This trend aligns with the evolution of more sophisticated methodologies over time. Geographically, no significant outcome differences were observed among Asia, Europe, and North America. Diverging performance metrics were evident when analyzing different tumor types. One possible explanation for these variations is tumor size. Multiple studies^33^^,^^45^ have indicated that smaller lesions generally yield lower segmentation accuracy. Because metastases are typically smaller, this could account for the commonly observed diminished segmentation accuracy. However, we needed to find a way to delve deeper into the relationship between tumor size and segmentation accuracy due to inconsistencies in size metrics reported across studies.

An intriguing observation emerged when considering all validation data sets, both internal and external: subgroup analyses did not reveal significant disparities in either segmentation or detection tasks. This might imply the commendable generalizability of AI models. However, this observation might also stem from including meticulously chosen high-quality research. Model construction errors can still lead to overfitting. Moreover, from the studies cited, external validation sets^29^^,^^30^^,^^33^^,^^37^^,^^41^^,^^43^ predominantly shared similar MRI manufacturer and model distributions. Only a single study^40^ with distinct MRI parameters displayed substantially inferior results. Our exploratory 3-level meta-analysis, clustered by data set, highlighted significant intra–data set variance and moderate inter–data set variance for all algorithms on all validation data sets. This suggests that although data set diversity might induce discrepancies that affect model performance, there may be more effective approaches than clustering based solely on data sets. A future direction might involve quantifying data set diversity—potentially hinging on MRI hardware and other variables—into a diversity index. This could serve as a more refined clustering criterion for multilevel meta-analyses.

The significant variance detected within data sets by the 3-level meta-analysis prompted a deeper examination of algorithm-related technical factors. Supplemental Tables 8 and 9 (available online at https://www.mcpdigitalhealth.org/) highlight distinct factors influencing the performance of segmentation and detection models. This distinction is logical because segmentation delves into detailed tumor compartments, whereas detection primarily aims to identify anomalous entities. In the context of segmentation, there was a noticeable difference among groups based on model input dimension. This could be attributed to the lesser performance observed in the 2D/3D ensemble models, particularly in metastasis results.

Regarding algorithm types, nnU-net^47^ showcased a superior performance than U-net variants, only lagging behind U-net and general CNNs. This order of performance seems logical, given the chronological development of these algorithms. When evaluating preprocessing methods, almost all improved outcomes within their respective subgroups. In particular, N4 bias field correction, data augmentation, and intensity normalization demonstrated statistically significant improvements. Skull stripping was also noteworthy, with borderline significance (*P*=.05). However, pixel resampling did not exhibit a significant difference among subgroups. This might be due to the different resolutions required between the x-y and z planes. This suggests that a more intricate engineering approach than a simple resampling method might be essential, as indicated in our previous study.^48^ The practice of cropping images, often used owing to computer memory constraints, appeared to have no substantial influence on the results.

In the realm of detection tasks, it was observed that lesion-wise sensitivity showed a strong correlation with the number of lesions incorporated during the training phase. However, regarding input dimensionality, 3D models consistently outperformed their 2D and 2D ensemble counterparts. This superior performance could likely be attributed to the richer spatial context provided by 3D models. Further differences emerged when examining the performance across various algorithmic approaches. Notably, despite its reputation for segmentation excellence, the nnU-net did not dominate detection tasks. Instead, U-net variants and traditional CNNs demonstrated superior results over U-net and nnU-net. This disparity emphasizes that a model’s specialization, such as segmentation, might only sometimes guarantee top-tier performance in diverse tasks. Finally, although preprocessing techniques are often lauded for their potential to enhance model performance, this was not the case for detection tasks. Specific techniques such as pixel resampling and intensity normalization were counterproductive. This observation suggests that specific preprocessing methods may inadvertently introduce artifacts or strip away crucial data, compromising the model’s detection abilities.

Integrating advanced MRI-based tumor detection and segmentation models holds immense promise for enhancing clinical care. Enhanced detection capabilities, particularly for smaller tumors, pave the way for early and more accurate diagnoses, often leading to improved patient outcomes. The precision afforded by accurate tumor segmentation is invaluable for defining tumor boundaries for surgical resections and radiation therapy planning and as a precursor for radiomics feature extraction. When used with radio genomics, this detailed segmentation mask could propel personalized medicine, tailoring treatments based on individual patient profiles. Furthermore, the consistent and efficient analysis of periodic scans ensures meticulous monitoring of tumor progression. As these models become more adaptable across diverse MRI protocols and machines, we inch closer to a future where patients globally receive uniform, high-quality care, regardless of the specific equipment.

Further research is imperative to enhance our understanding of how diverse data sets, such as demographic details and MRI hardware specifications, impact the performance of AI models. A comprehensive study encompassing several data sets with intricate details about demographics, MRI hardware, and other potential factors could pave the way to quantifying a diversity index for data sets. Moreover, meta-analysis focused on specific brain tumors such as glioblastoma, meningioma, or brain metastases is essential to understand better the performance of DL models in a clinical setting. Moreover, larger meta-analyses, focusing on various tumor types and tasks, are warranted to boost statistical power when analyzing algorithm-related technical factors. Finally, exploring innovative techniques such as harmonization methods or generative adversarial networks holds promise. Such strategies could generate more diverse data, augmenting the generalization capabilities of AI in clinical applications.

## Conclusion

This systematic review and meta-analysis delved into the factors contributing to data set diversity and its impact on brain tumor detection and segmentation models. Our findings underscored the pivotal role of MRI hardware and brain tumor type in shaping data set diversity. Meanwhile, the technical intricacies underlying AI construction also emerged as significant determinants of model performance. Although enhanced MRI-based detection and segmentation models hold transformative potential for patient care, our results illuminate areas needing further exploration. The quest for a comprehensive diversity index for data sets amplified meta-analyses and innovative techniques such as generative adversarial networks for data diversification remain on the horizon. As the medical landscape navigates these promising terrains, rigorous research and AI application synergy can herald a transformative era in oncological patient care.

## Potential Competing Interests

The authors declare that they have no known competing financial interests or personal relationships that could have appeared to influence the work reported in this article.
