# Supplementary material for: Artificial Intelligence Detection and Segmentation Models: A Systematic Review and Meta-Analysis of Brain Tumors in Magnetic Resonance Imaging
Source: Mayo Clin Proc Digit Health. 2024 Feb 4;2(1):75–91. doi: 10.1016/j.mcpdig.2024.01.002 (PMC11976016; doi:10.1016/j.mcpdig.2024.01.002)
Supplement: Supplementary Materials [file mmc1.docx]

Supplementary Materials: Artificial intelligence Detection and Segmentation Models: A Systematic Review and Meta-analysis of Brain Tumors in MRI

Ting-Wei Wang , Yu-Chieh Shiao, Jia-Sheng Hong, Wei-Kai Lee, Ming-Sheng Hsu, Hao-Min Cheng, Huai-Che Yang , Cheng-Chia Lee, Hung-Chuan Pan, Weir Chiang You, Jiing-Feng Lirng, Wan-Yuo Guo , Yu-Te Wu*

**
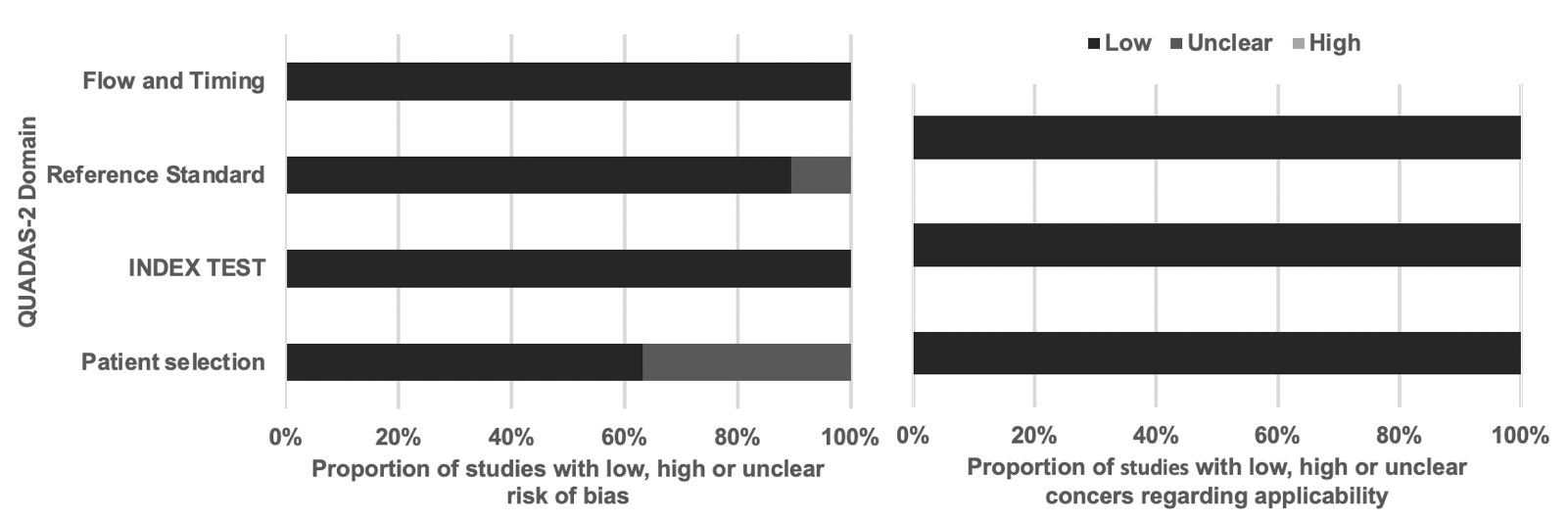
**

**Figure S1. The results of QUADAS-2 quality assessment for included studies**

**
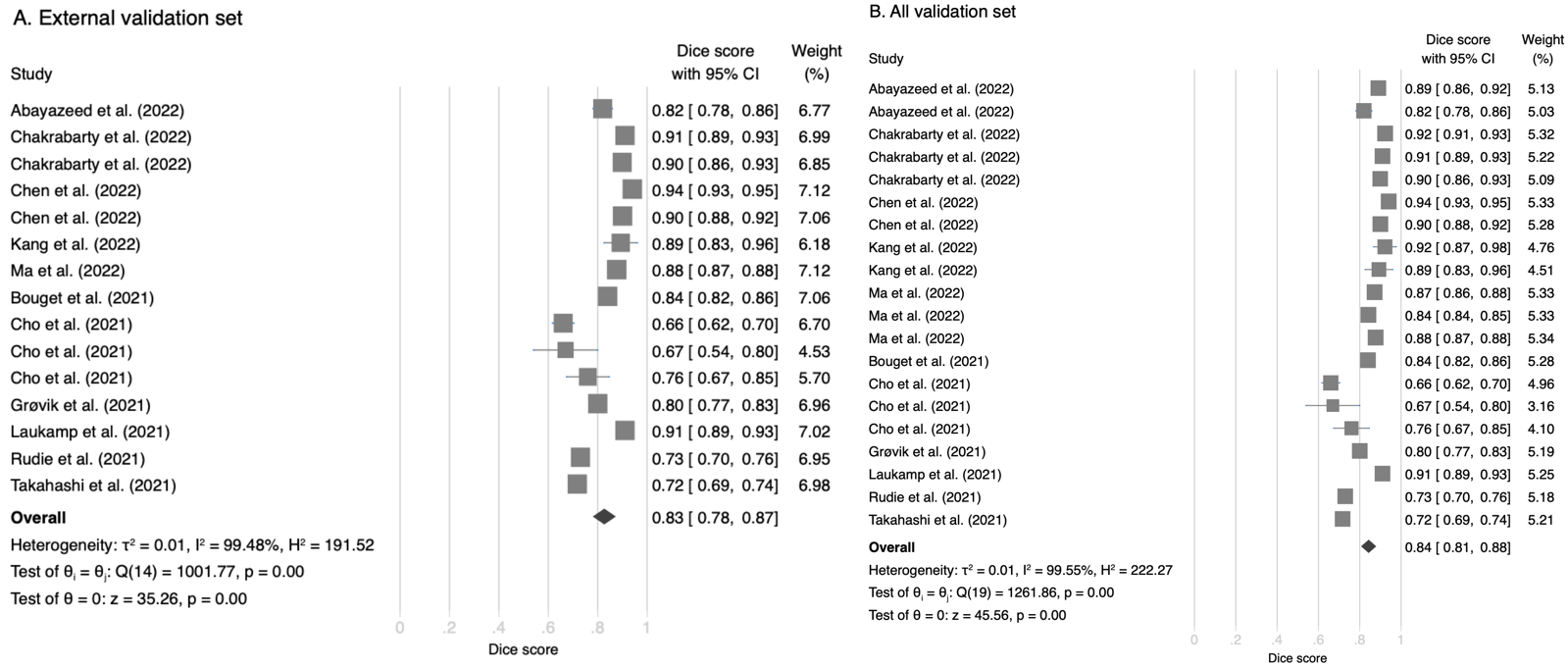
**

**Figure S2. Forest plot of deep learning algorithms’ lesion-wise dice score restricted to algorithms reporting the highest accuracy**

**
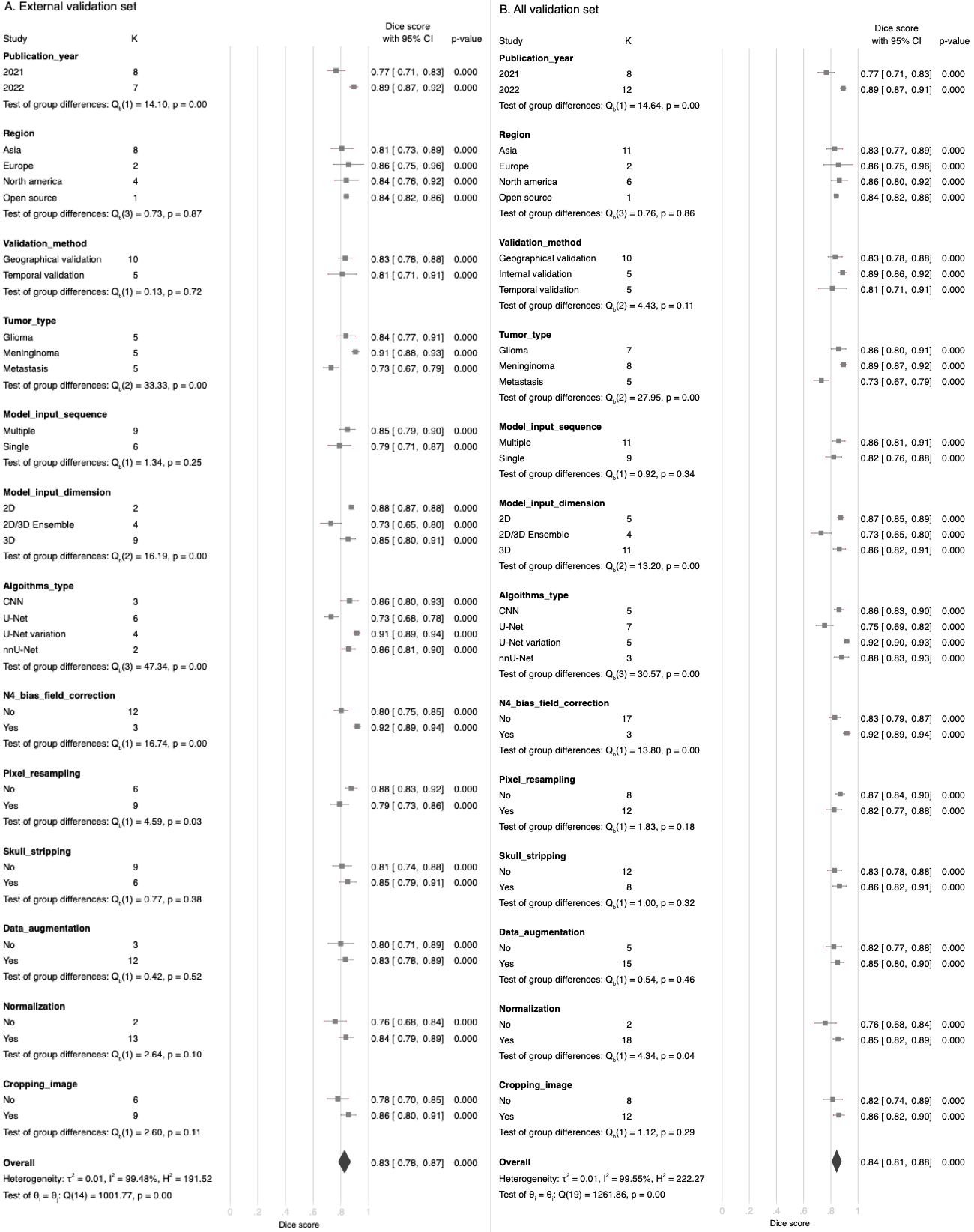
**

**Figure S3. Forest plot of subgroup analysis of deep learning algorithms’ lesion-wise dice score restricted to algorithms reporting the highest accuracy**

**
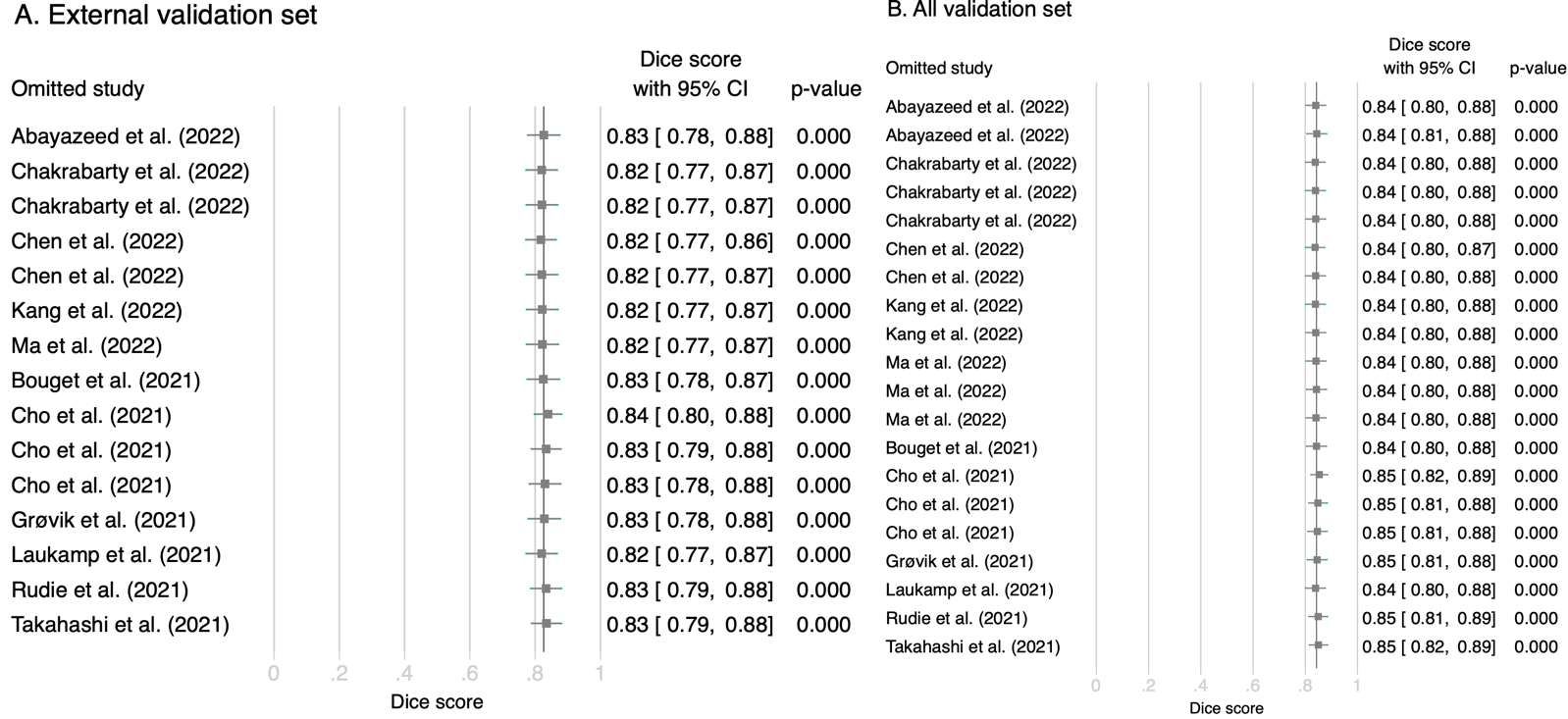
**

**Figure S4. The results of a sensitivity analysis of deep learning algorithms’ lesion-wise dice score restricted to algorithms reporting the highest accuracy using the one-study removal method**

**
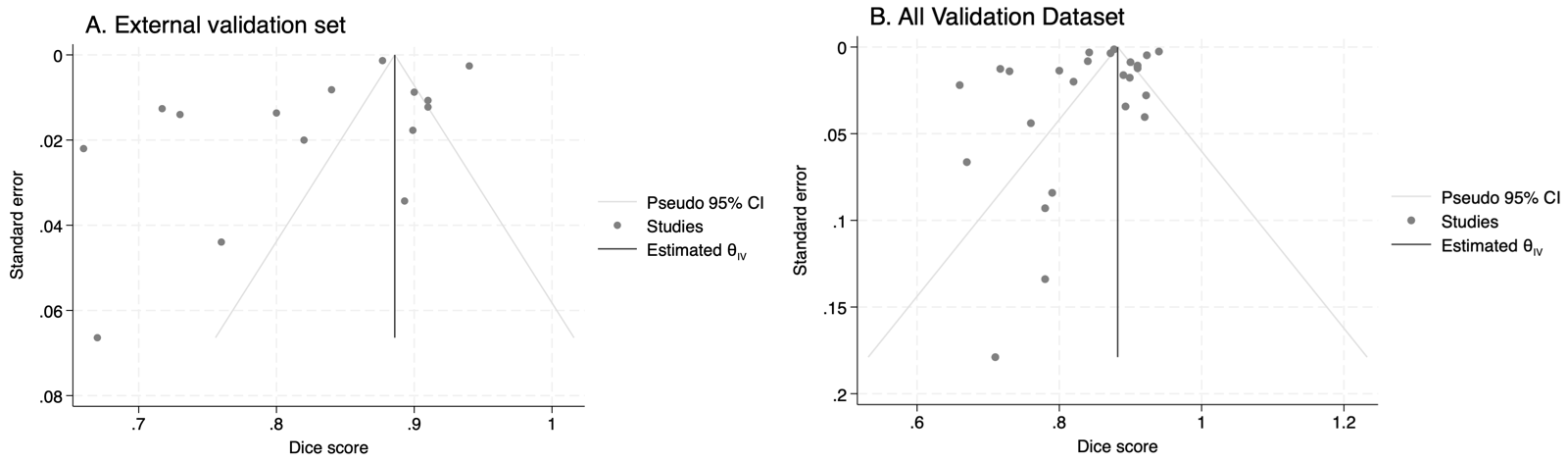
**

**Figure S5. The funnel plot of deep learning algorithms’ lesion-wise dice score restricted to algorithms reporting the highest accuracy** The *p* value of the Egger's test was 0.0397 for external validation dataset, 0.03388 for all validation dataset, indicating present of publication bias.

**
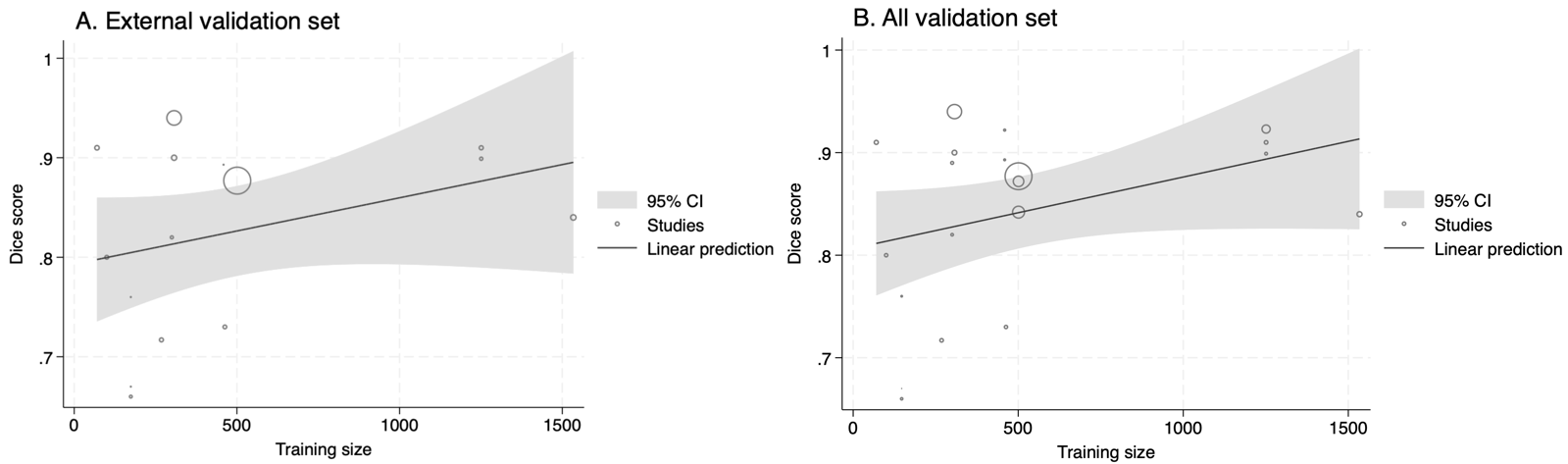
**

**Figure S6. Bubble plot of meta regression of deep learning algorithms’ lesion-wise dice score restricted to algorithms reporting the highest accuracy on training size** A. External validation dataset: training size (Coefficient: 0.0000667, p=0.187). B. All validation dataset: (Coefficient: 0.0000696, p=0.090)


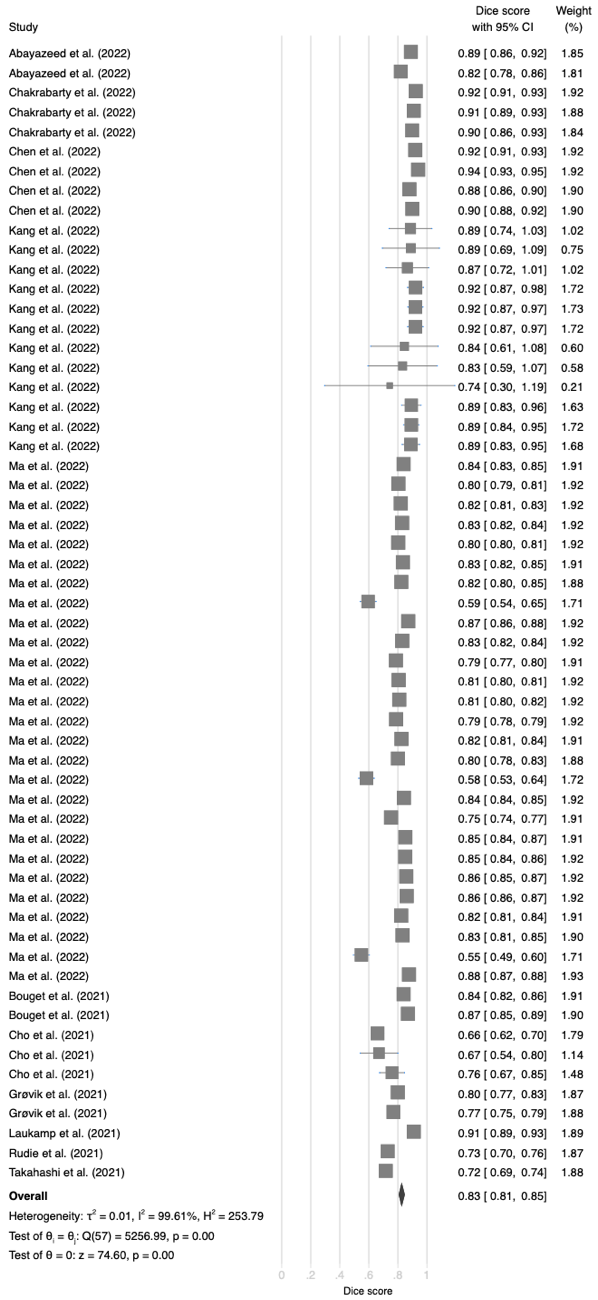


**Figure S7. Forest plot of deep learning algorithms’ lesion-wise dice score with all reported algorithms on all validation dataset**

**
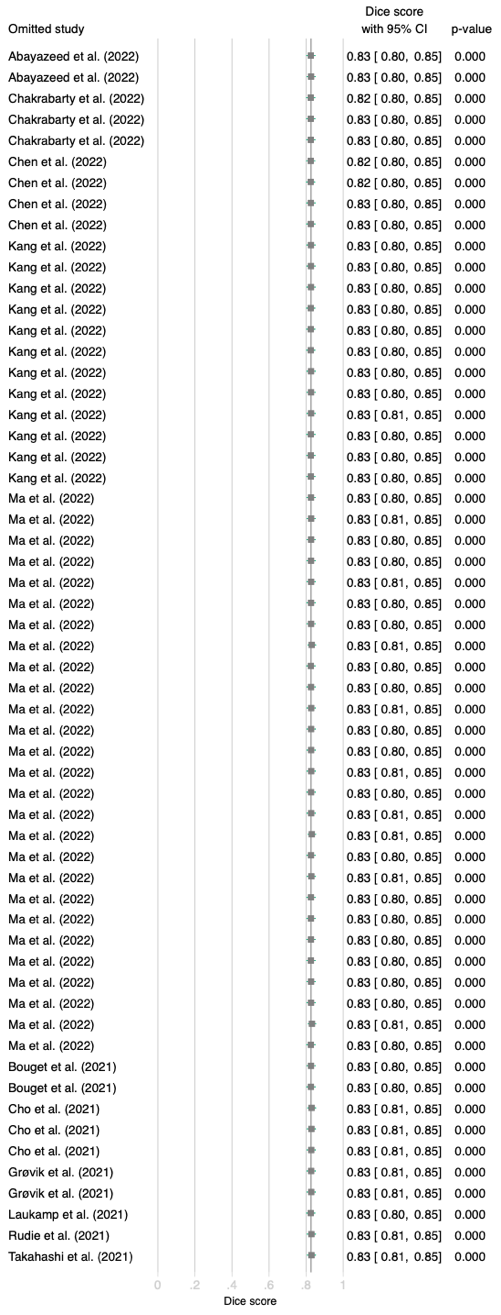
**

**Figure S8. The results of a sensitivity analysis of deep learning algorithms’ lesion-wise dice score with all reported algorithms on all validation dataset using the one-study removal method**

**
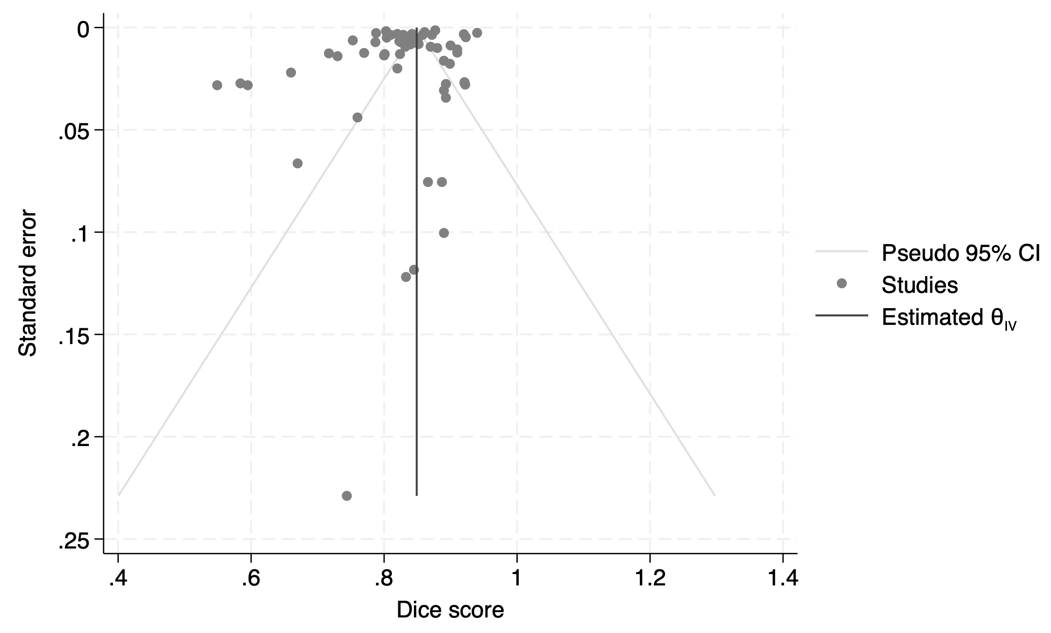
**

**Figure S9. The funnel plot of deep learning algorithms’ lesion-wise dice score with all reported algorithms on all validation dataset.** The *p* value of the Egger's test was 0.3978 indicating no publication bias.

**
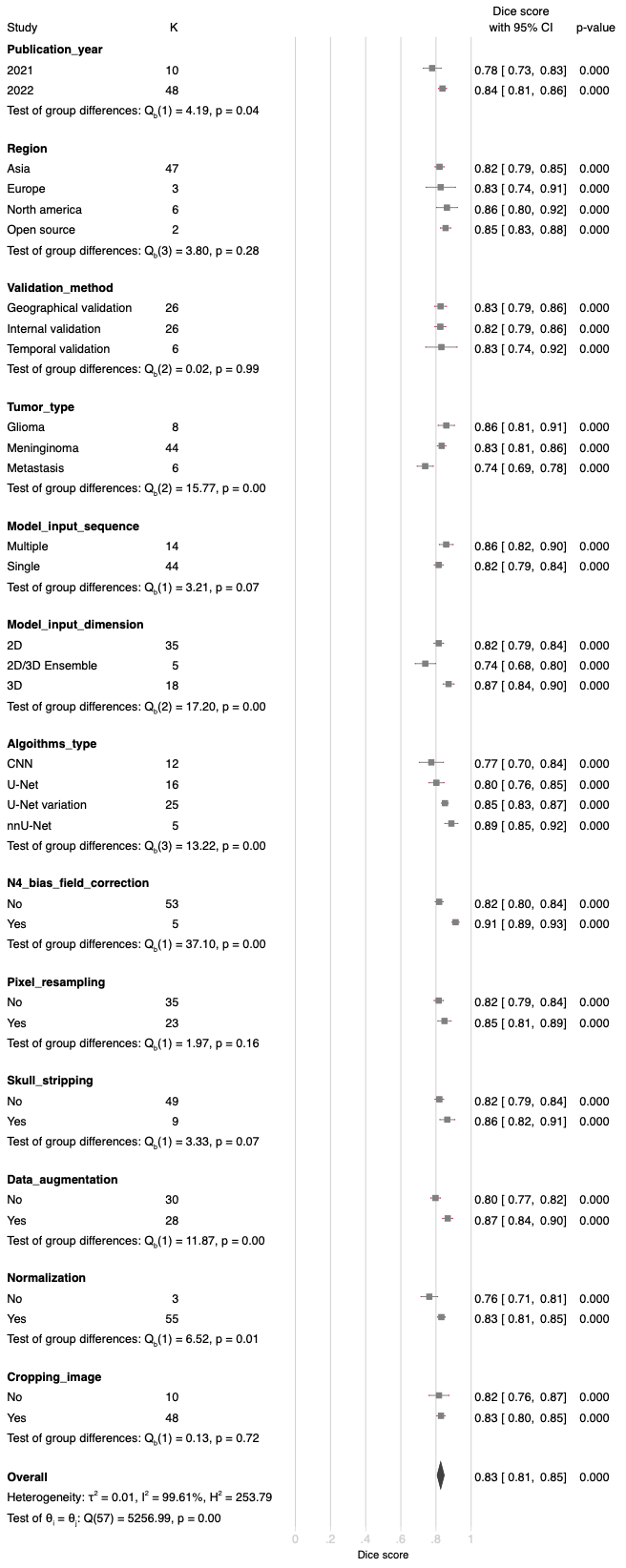
**

**Figure S10. Forest plot of subgroup analysis of deep learning algorithms’ lesion-wise dice score with all reported algorithms on all validation dataset**

**
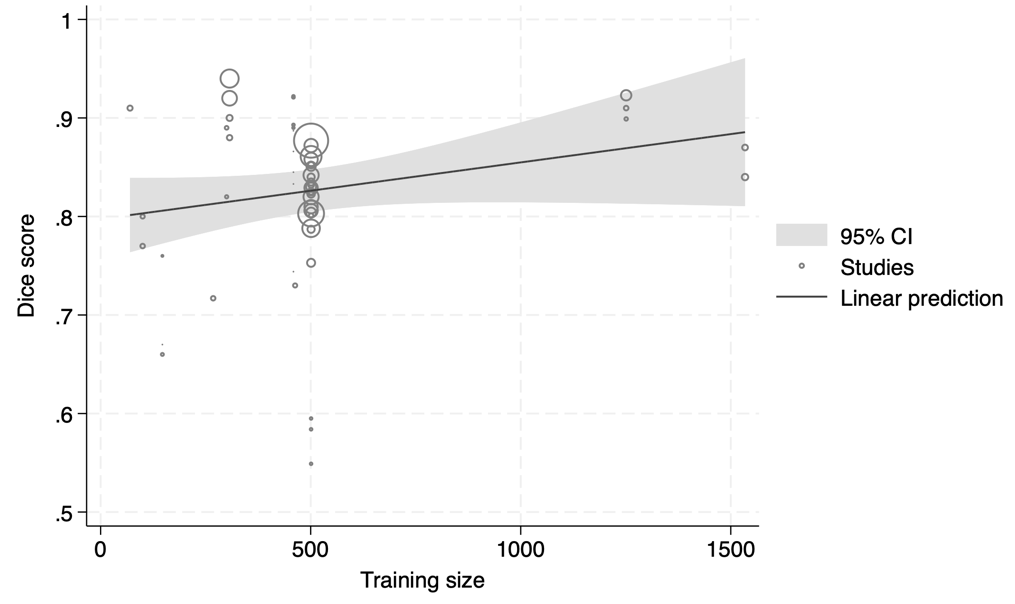
**

**Figure S11. Bubble plot of meta regression of deep learning algorithms’ lesion-wise dice score with all reported algorithms on all validation dataset on training size** (Coefficient: 0.0000575, p=0.109).

**
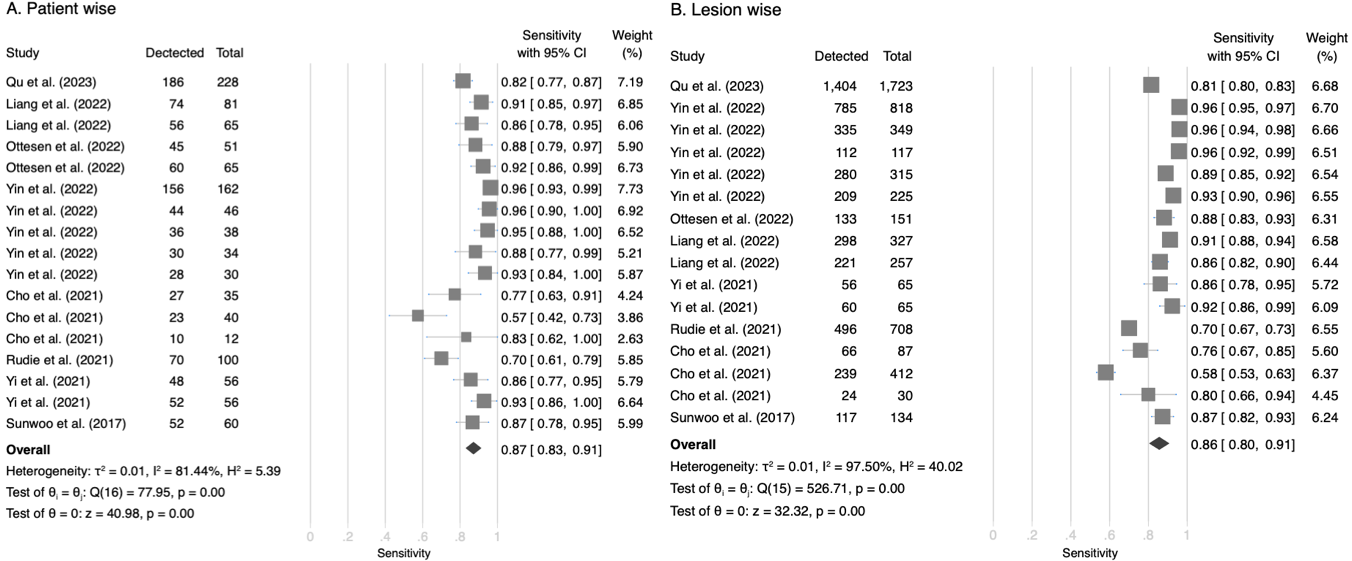
**

**Figure S12. Forest plot of algorithms’ sensitivity restricted to algorithms reporting the highest accuracy on all validation dataset**


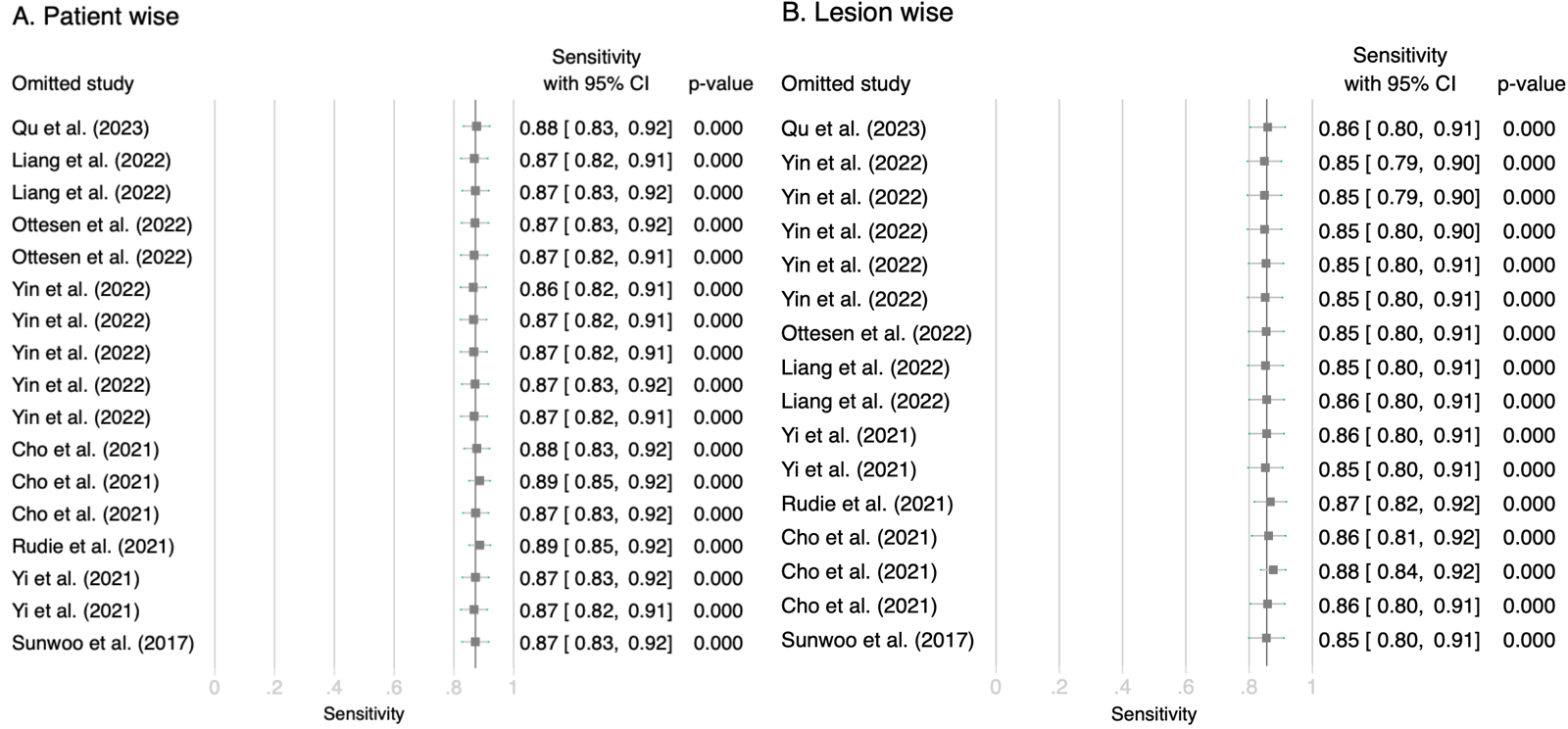


**Figure S13. The results of a sensitivity analysis of algorithms’ sensitivity restricted to algorithms reporting the highest accuracy on all validation dataset using the one-study removal method**


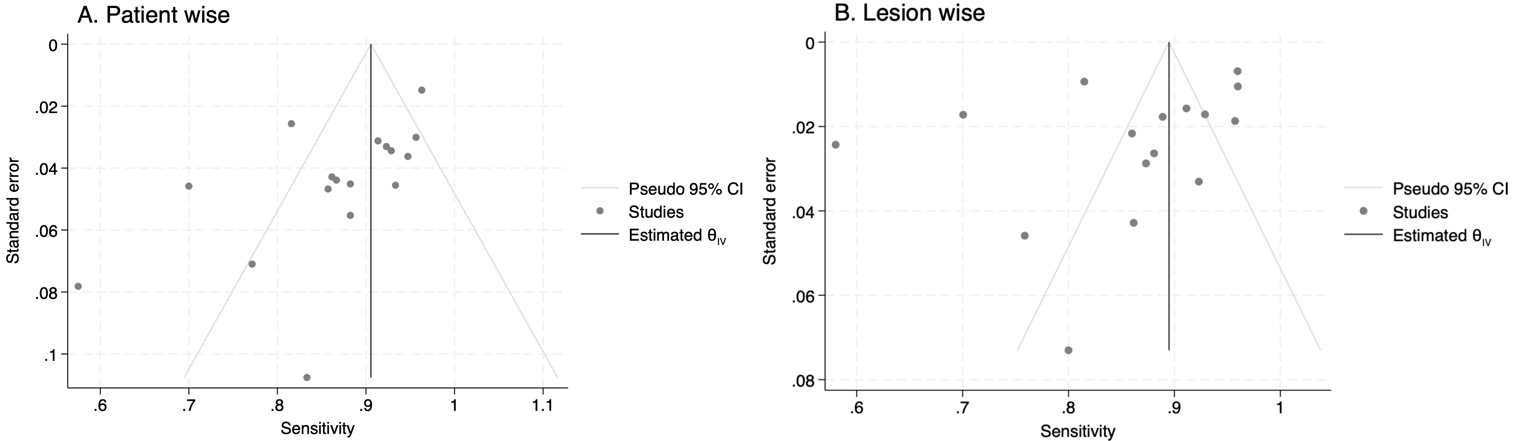


**Figure S14. The funnel plot of algorithms’ sensitivity restricted to algorithms reporting the highest accuracy on all validation dataset** The *p* value of the Egger's test was 0.0029 for patient wise sensitivity, indicating present of publication bias; 0.0951 for lesion wise sensitivity, indicating no publication bias.


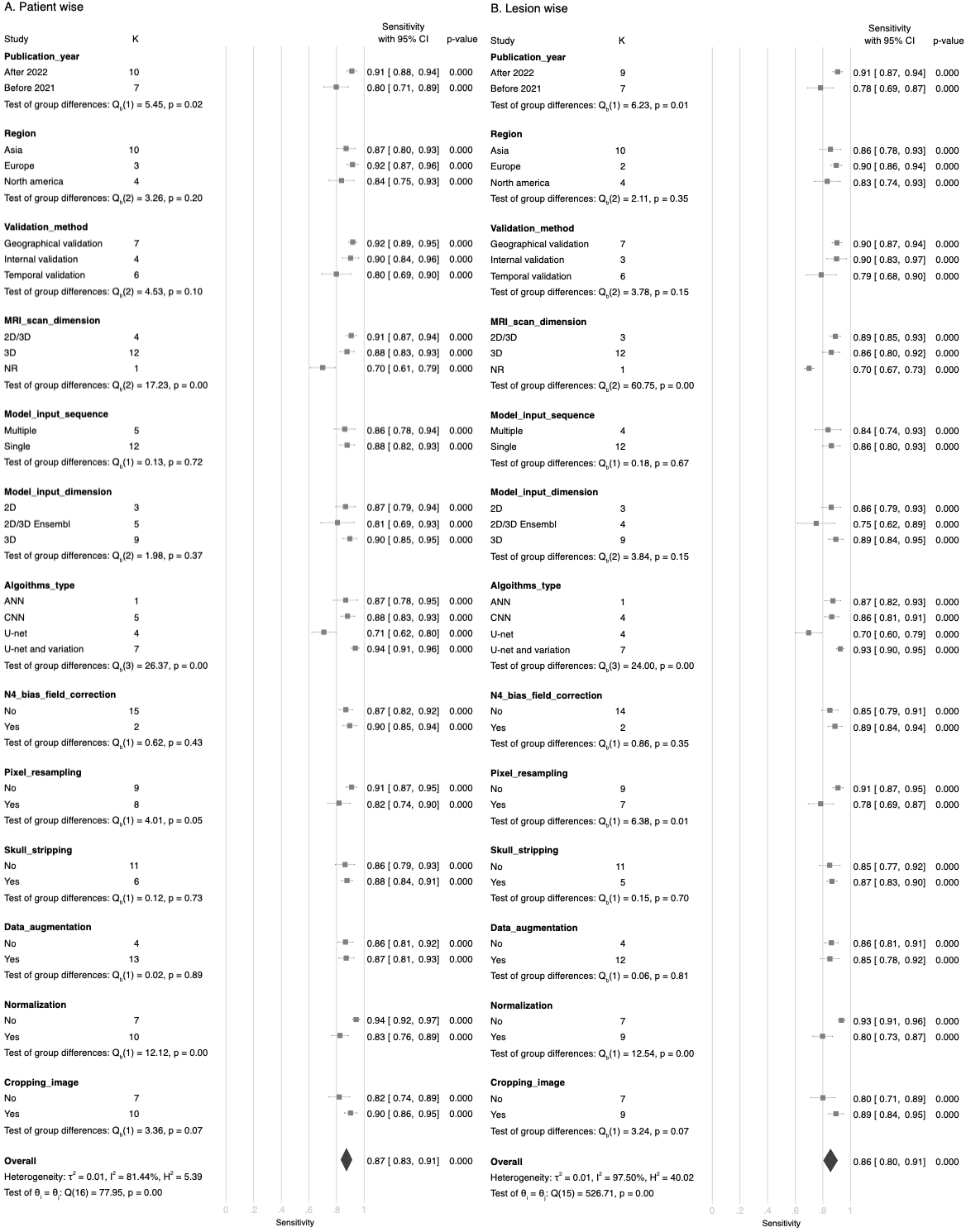


**Figure S15. Forest plot of subgroup analysis of algorithms’ sensitivity restricted to algorithms reporting the highest accuracy on all validation dataset**


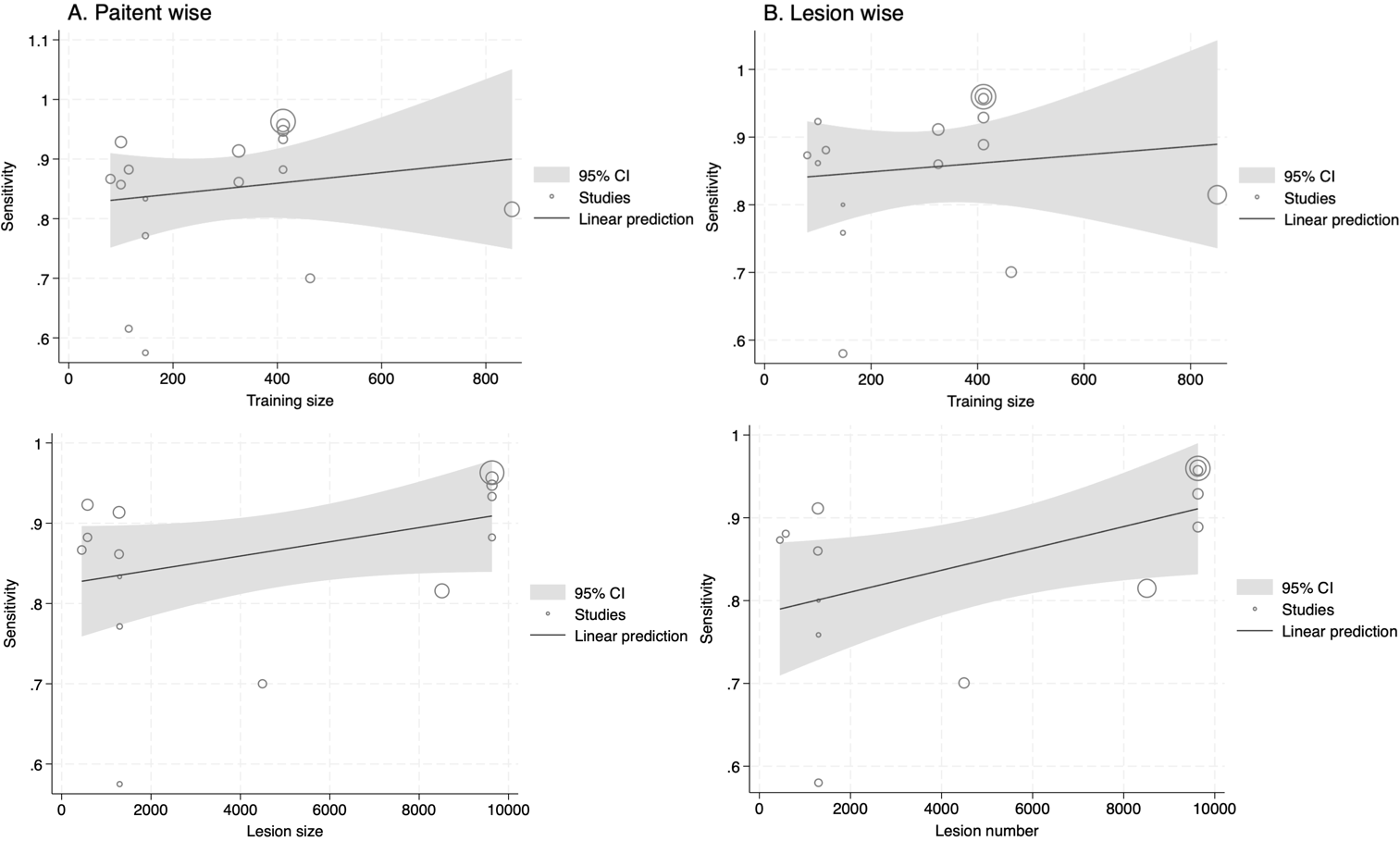


**Figure S16. Bubble plot of meta regression of algorithms’ sensitivity restricted to algorithms reporting the highest accuracy on all validation dataset on Training size and lesion numbers** A. Patient wise sensitivity: train size (Coefficient: 7.82e-7, p=0.994); lesion number Coefficient: 8.86e-6, p=0.122) B. Lesion wise sensitivity: train size (Coefficient: 0.0000534, p=0.731); lesion number (Coefficient: 0.0000131, p=0.089)


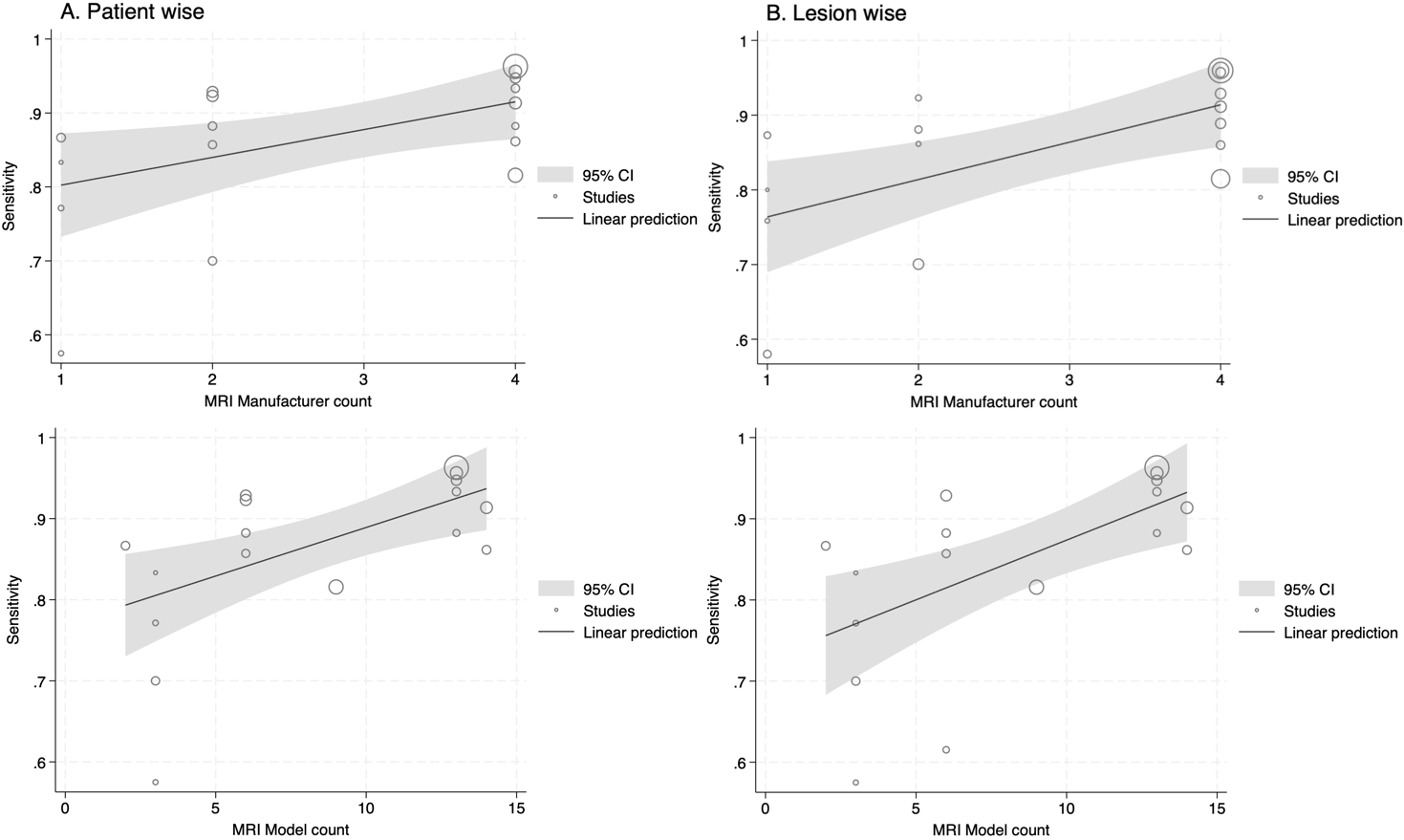


**Figure S17. Bubble plot of meta regression of algorithms’ sensitivity restricted to algorithms reporting the highest accuracy on all validation dataset on MRI manufacturer and MRI model count** A. Patient wise sensitivity**:** MRI manufacturer (Coefficient: .0375866, p=0.017*); MRI model count (Coefficient: .0119754, p=0.002*) B. Lesion wise sensitivity: MRI manufacturer (Coefficient: 0.0000534, p=0.731); MRI model count (Coefficient: 0.0000131, p=0.089)

**
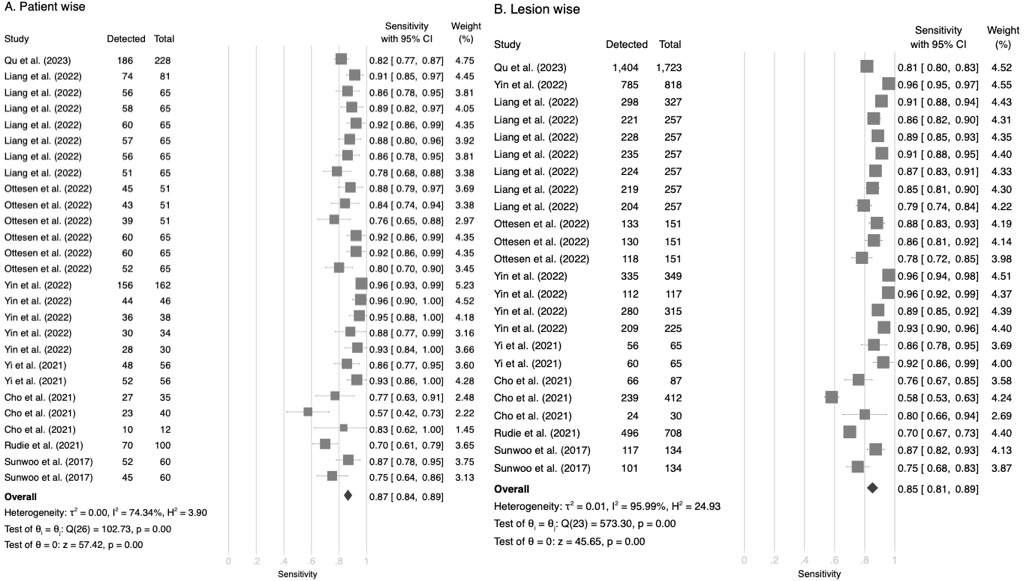
**

**Figure S18. Forest plot of** **algorithms’ sensitivity with all algorithms reporting the highest accuracy on external validation dataset**

**
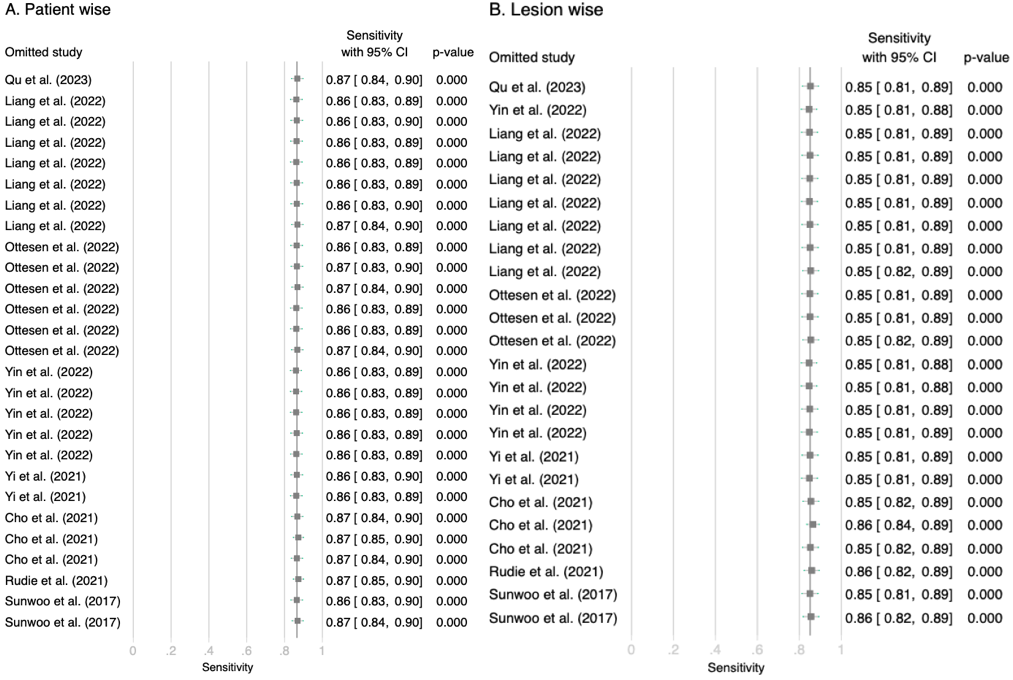
**

**Figure S19. The results of a sensitivity analysis of algorithms’ sensitivity with all algorithms reporting the highest accuracy on external validation dataset using the one-study removal method**

**
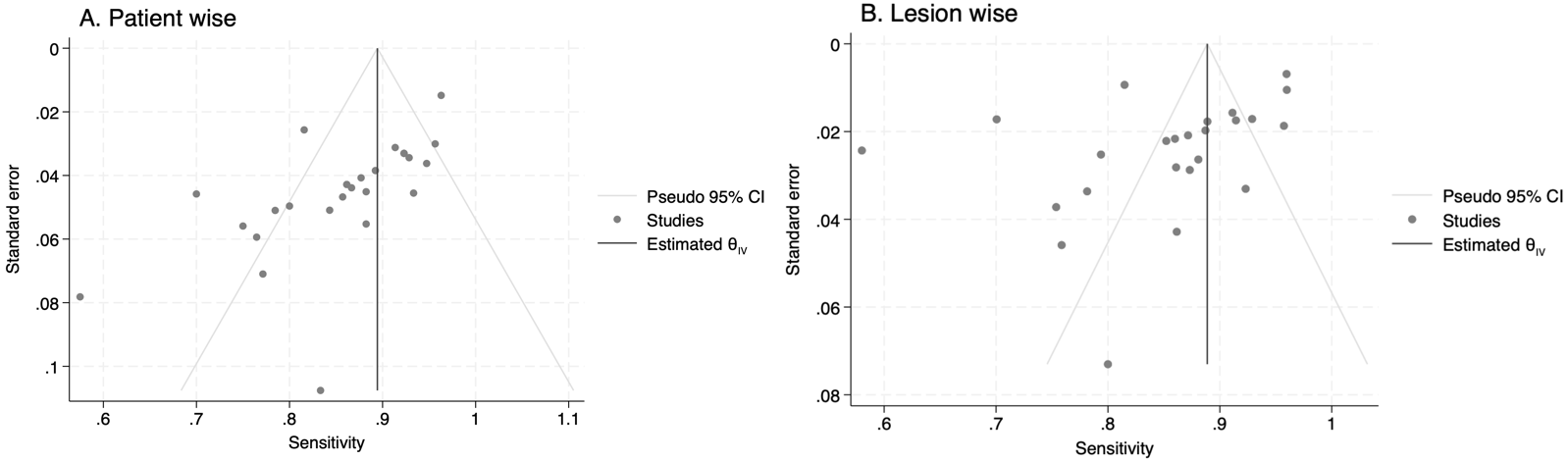
**

**Figure S20. The funnel plot of algorithms’ sensitivity with all algorithms reporting the highest accuracy on external validation dataset.** The *p* value of the Egger's test was 0.0267 for patient wise sensitivity, indicating present of publication bias; 0.4477 for lesion wise sensitivity, indicating no publication bias.

**
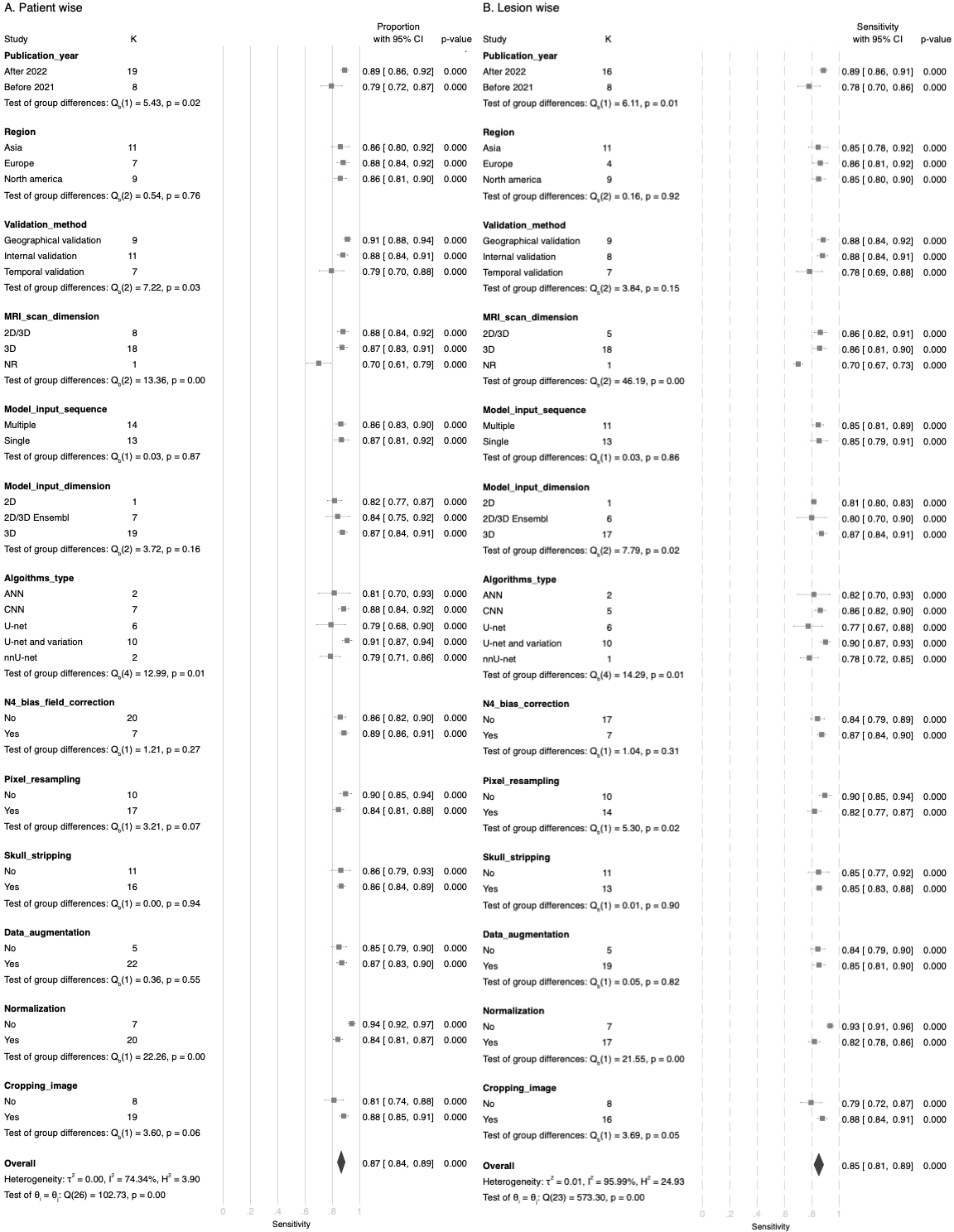
**

**Figure S21. Forest plot of subgroup analysis of algorithms’ sensitivity with all algorithms reporting the highest accuracy on external validation dataset**

**
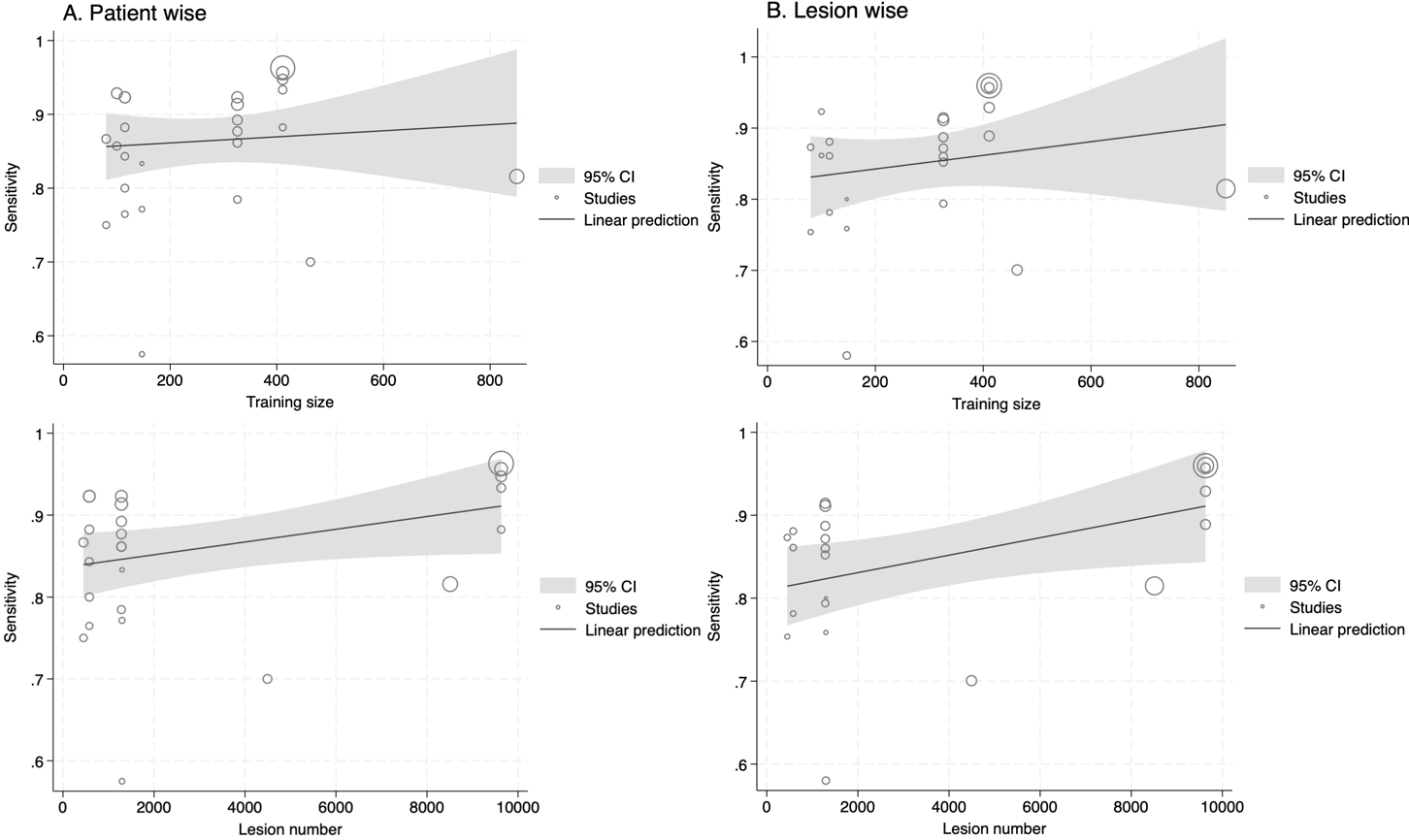
**

**Figure S22. Bubble plot of meta regression of algorithms’ sensitivity with all algorithms reporting the highest accuracy on external validation dataset on Training size and lesion size**

A. Patient wise sensitivity: train size (Coefficient: -6.73e-06, p=0.961); lesion number Coefficient: 9.51e-06 p=0.202) B. Lesion wise sensitivity: train size (Coefficient: 0.0000534, p=0.731); lesion number (Coefficient: 0.0000131, p=0.089)

**
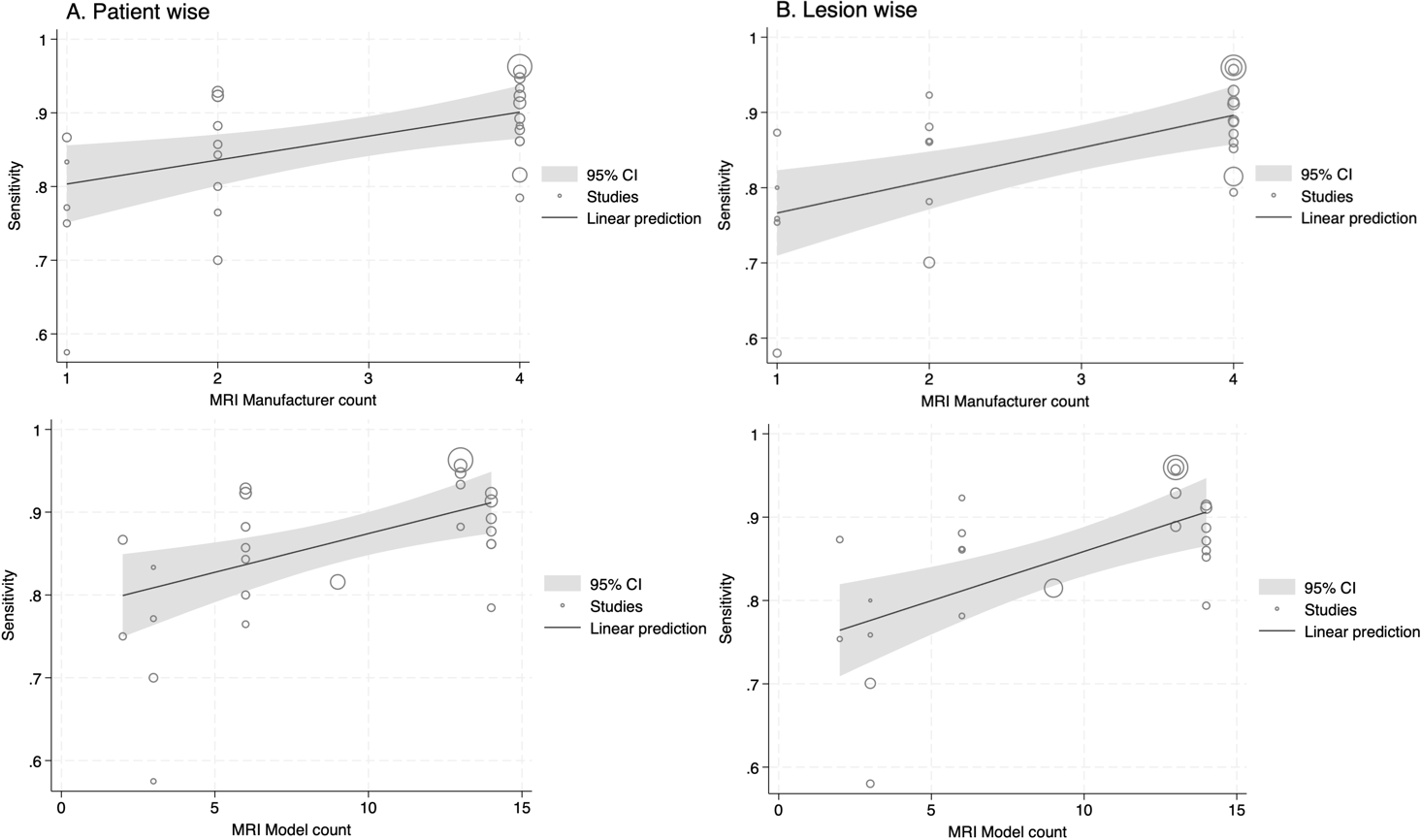
**

**Figure S23. Bubble plot of meta regression of algorithms’ sensitivity with all algorithms’ sensitivity with all algorithms reporting the highest accuracy on external validation dataset on MRI manufacture and MRI model count** A. Patient wise sensitivity: MRI Manufacture count (Coefficient: .0005716, p=0.983); MRI Model count (Coefficient: .0078015, p=0.376) B. Lesion wise sensitivity: MRI Manufacture count (Coefficient: 0.004172, p=0.891); MRI Model count (Coefficient: 0.0092474 p=0.354)

**
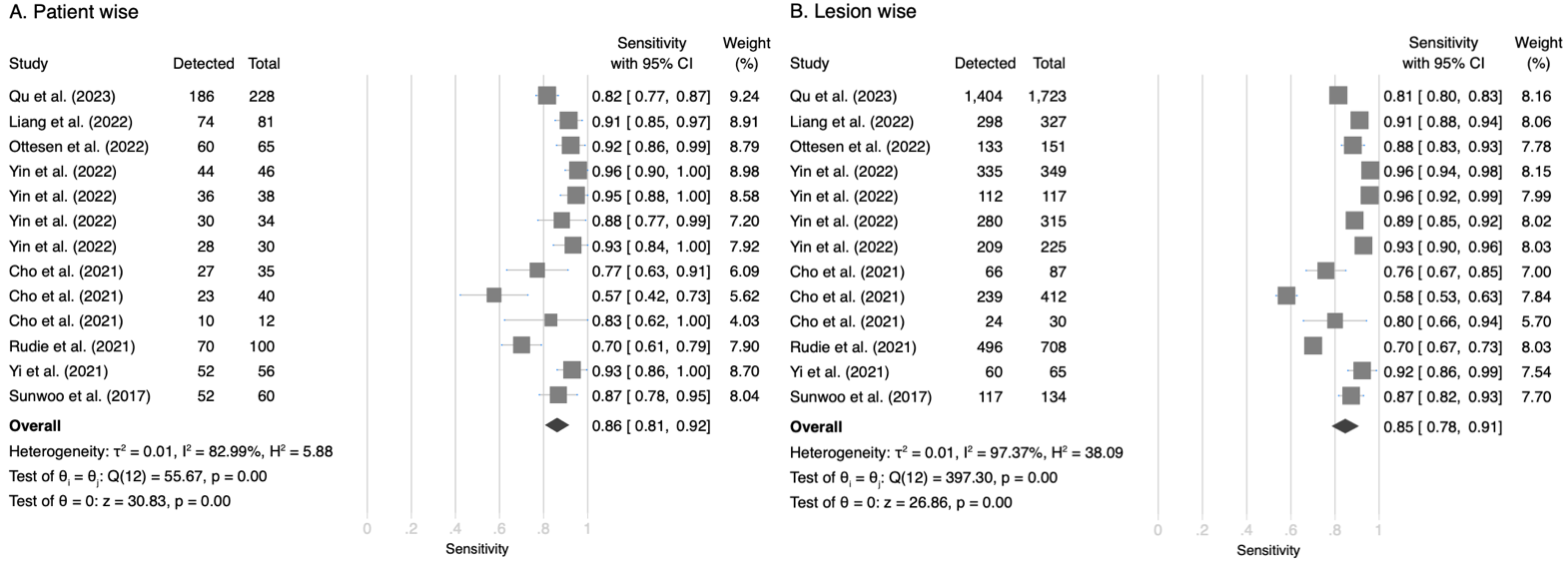
**

**Figure S24. Forest plot of** **algorithms’ sensitivity with all algorithms reporting on all validation dataset**


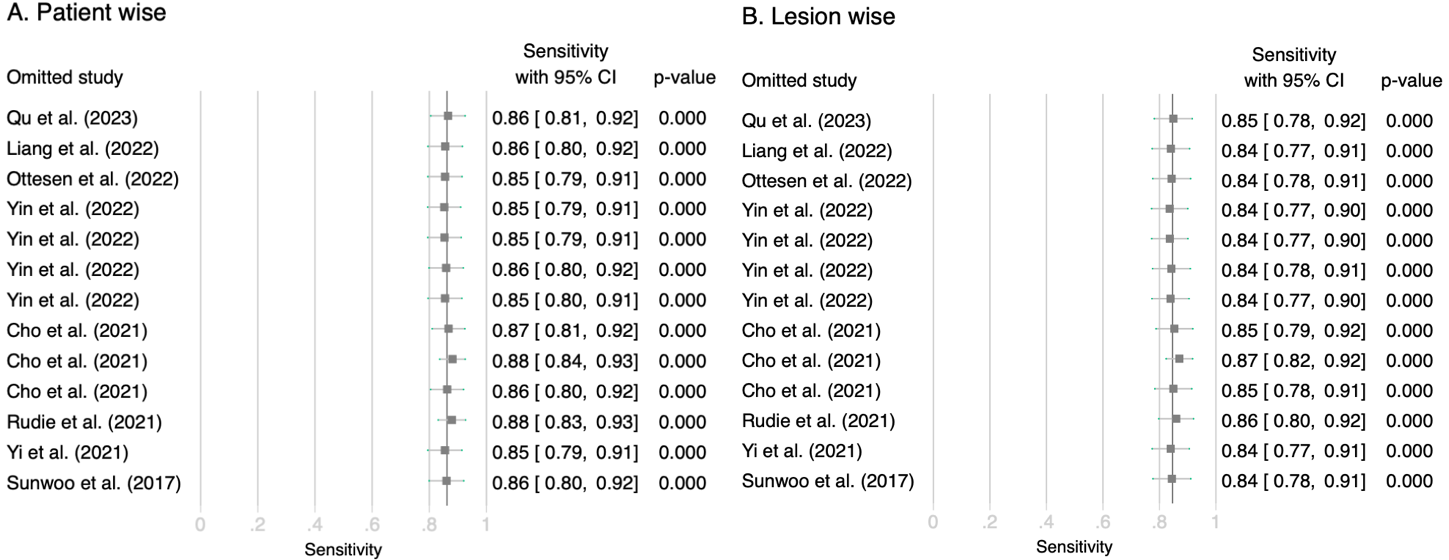


**Figure S25. The results of a sensitivity analysis of algorithms’ sensitivity with all algorithms reporting on all validation dataset using the one-study removal method**


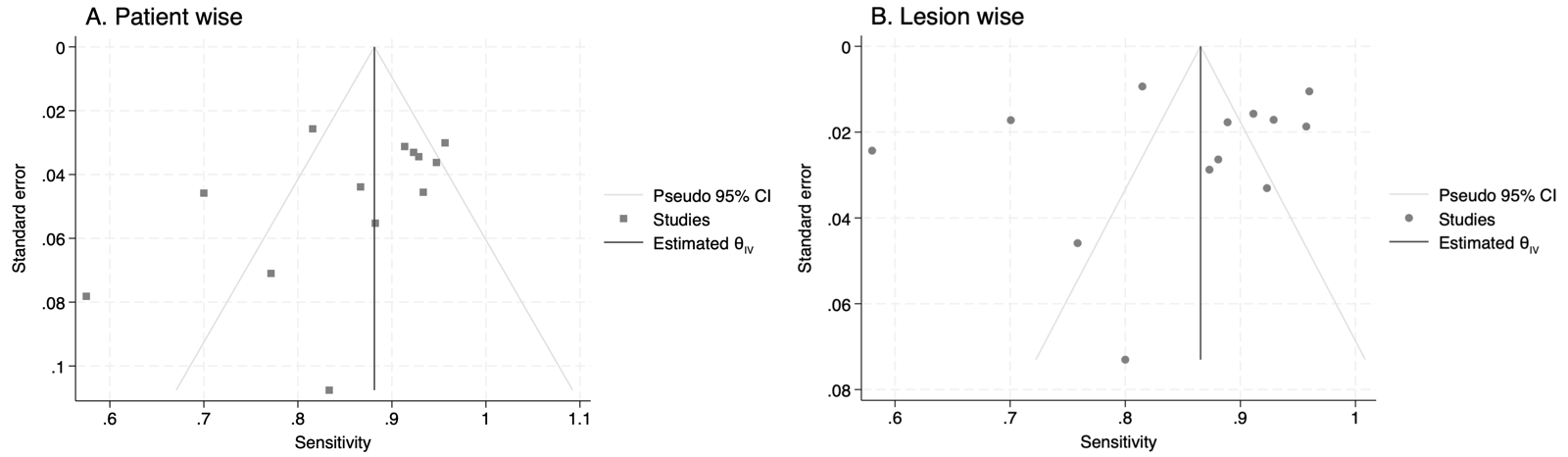


**Figure S26. The funnel plot of algorithms’ sensitivity with all algorithms reporting on all validation dataset.** The *p* value of the Egger's test was 0.0000 for patient wise sensitivity indicating present of publication bias., 0.0951 for all lesion wise sensitivity, indicating no publication bias.


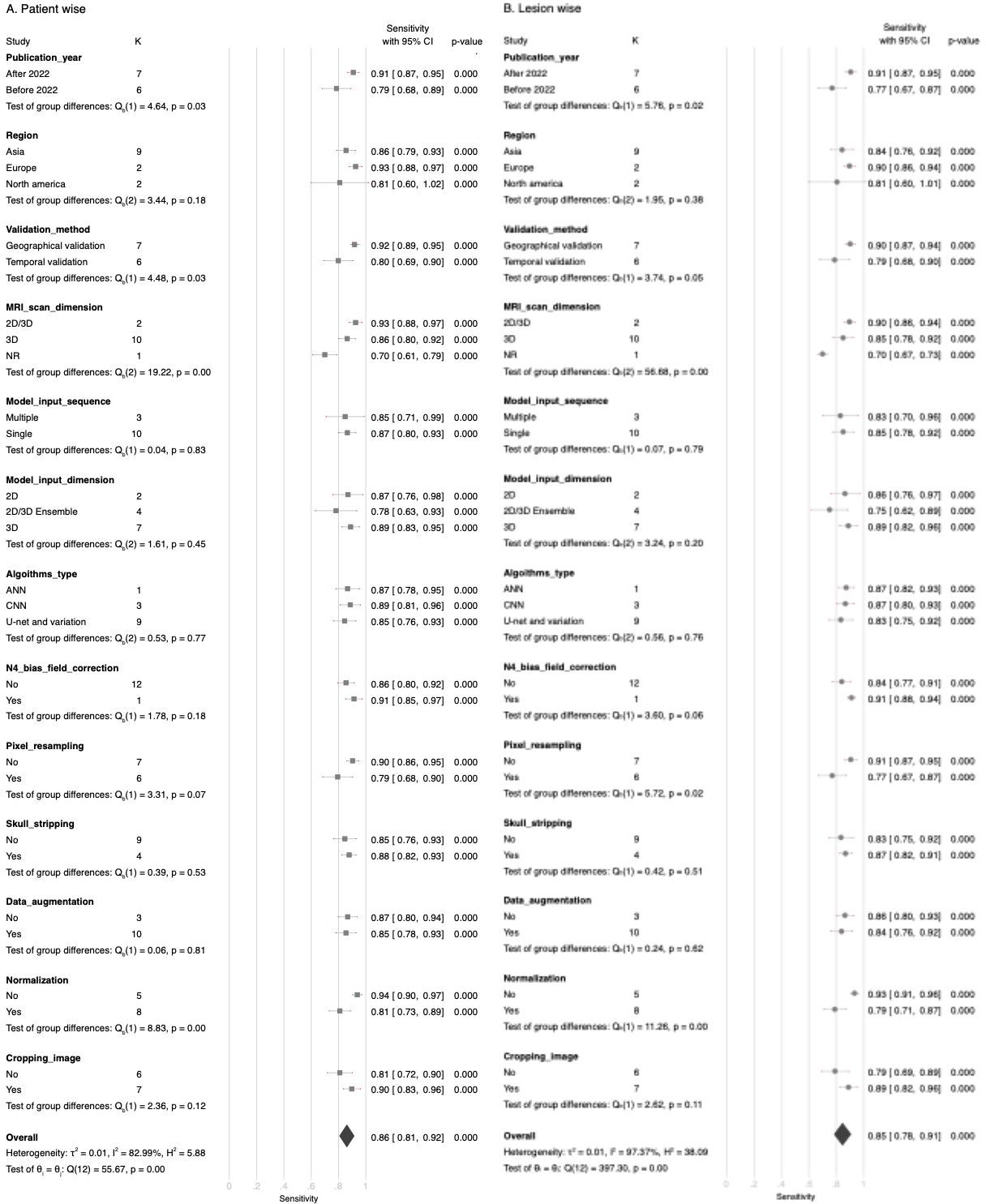


**Figure S27. Forest plot of subgroup analysis of algorithms’ sensitivity with all algorithms reporting on all validation dataset**


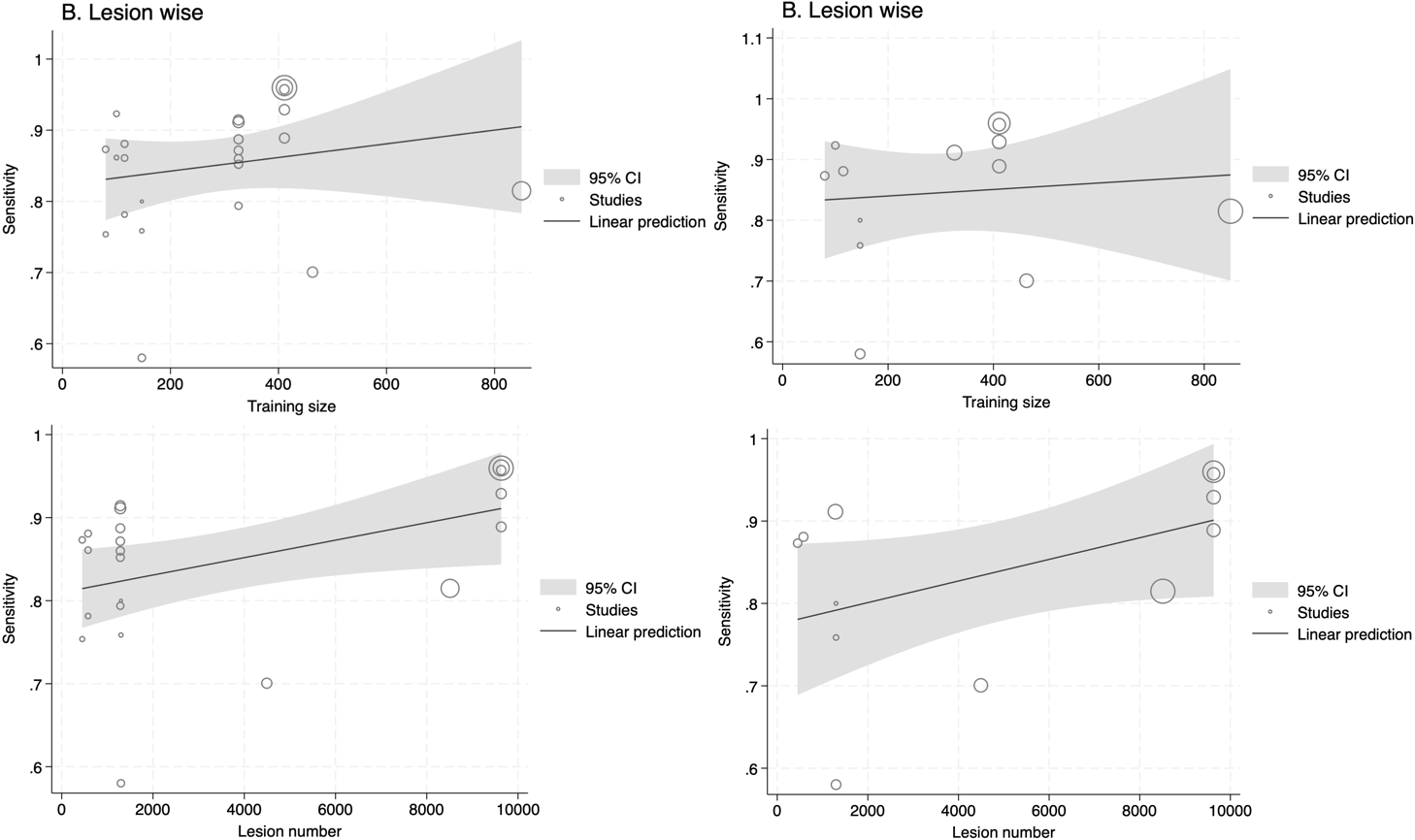


**Figure S28. Bubble plot of meta regression of algorithms’ sensitivity with all algorithms reporting on all validation dataset on Training size and lesion size** A. Patient wise sensitivity: train size (Coefficient: .0000412, p=0.628); lesion number Coefficient: 7.79e-06, p=0.056) B. Lesion wise sensitivity: train size (Coefficient: .0325202 , p=0.006); lesion number (Coefficient: .0093544, p=0.002)


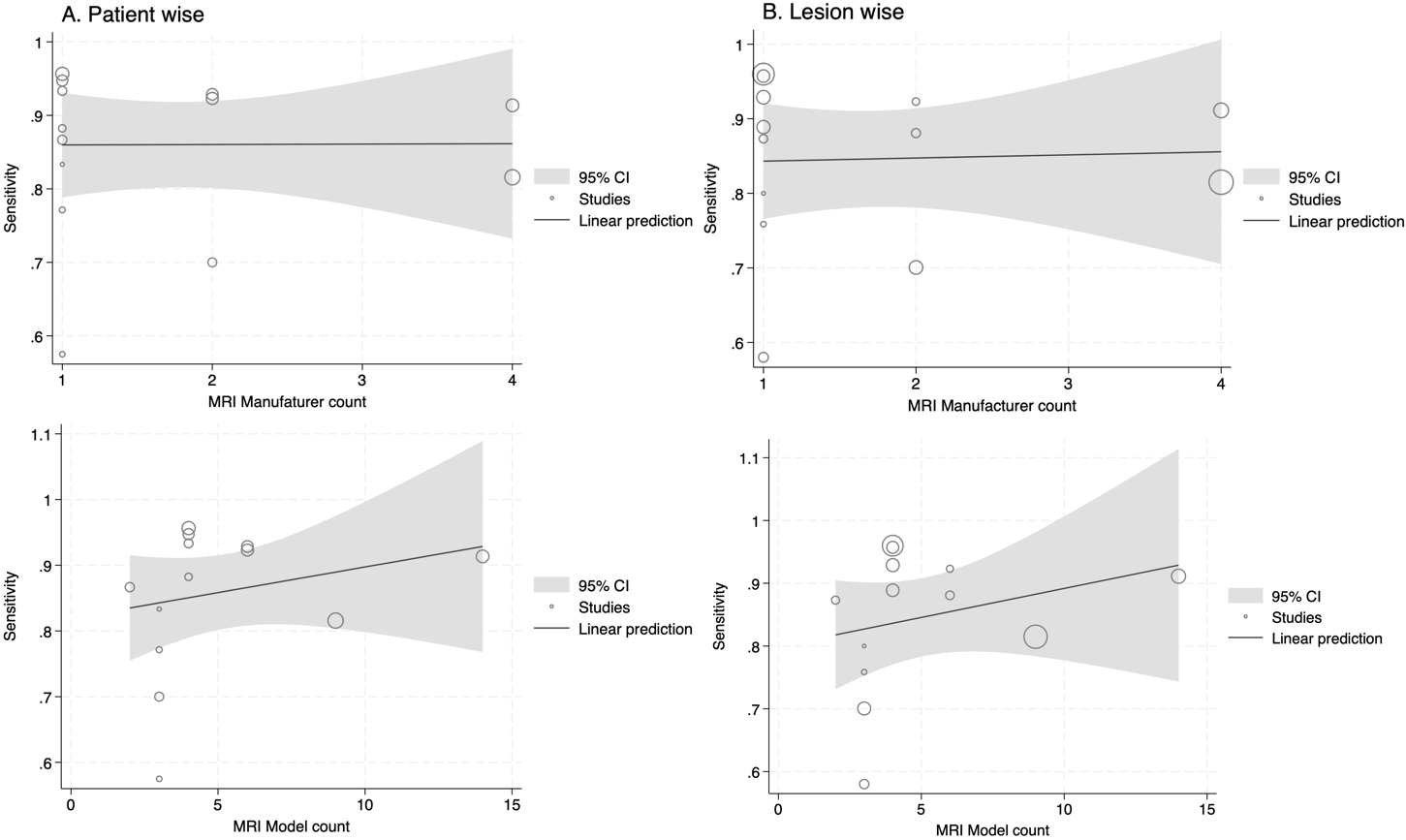


**Figure S29. Bubble plot of meta regression of algorithms’ sensitivity with all algorithms reporting on all validation dataset on MRI manufacture and MRI model count** A. Patient wise sensitivity: MRI Manufacture count (Coefficient: .0005716, p=0.983); MRI Model count (Coefficient: .0078015, p=0.376) B. Lesion wise sensitivity: MRI Manufacture count (Coefficient: 0.004172, p=0.891); MRI Model count (Coefficient: 0.0092474 p=0.354)

**Table S1. PRISMA-DTA Abstract Checklist.**

| **Section/Topic** | **Number** | **PRISMA-DTA for Abstracts Checklist Item** | **Reported on Page #** |
| --- | --- | --- | --- |
| TITLE and PURPOSE | | | |
| Title | 1 | Identify the report as a systematic review (+/- meta-analysis) of diagnostic test accuracy (DTA) studies. | 1 |
| Objectives | 2 | Indicate the research question, including components such as participants, index test, and target conditions. | 1 |
| METHODS | | | |
| Eligibility criteria | 3 | Include study characteristics used as criteria for eligibility. | 2 |
| Information sources | 4 | List the key databases searched and the search dates. | 2 |
| Risk of bias & applicability | 5 | Indicate the methods of assessing risk of bias and applicability. | 2 |
| Synthesis of results | A1 |  | 2 |
| RESULTS | | | |
| Included studies | 6 | Indicate the number and type of included studies and the participants and relevant characteristics of the studies (including the reference standard). | 2 |
| Synthesis of results | 7 | Include the results for the analysis of diagnostic accuracy, preferably indicating the number of studies and participants. Describe test accuracy including variability; if meta-analysis was done, include summary results and confidence intervals. | 2 |
| DISCUSSION | | | |
| Strengths and limitations | 9 | Provide a brief summary of the strengths and limitations of the evidence | 2 |
| Interpretation. | 10 | Provide a general interpretation of the results and the important implications. | 2 |
| OTHER | | | |
| Funding | 11 | Indicate the primary source of funding for the review | 2 |
| Registration | 12 | Provide the registration number and the registry name | NA |

Adapted From: McInnes MDF, Moher D, et al. The PRISMA-DTA Group (2018). Preferred Reporting Items for a Systematic Review and Meta-analysis of Diagnostic Test Accuracy Studies: The PRISMA-DTA Statement. JAMA. 2018 Jan 23;319(4):388-396. doi: 10.1001/jama.2017.19163.

**Table S2. PRISMA-DTA Checklist.**

| **Section/Topic** | **Number** | **PRISMA-DTA for Abstracts Checklist Item** | **Reported on Page #** |
| --- | --- | --- | --- |
| TITLE and PURPOSE | | | |
| Title | 1 | Identify the report as a systematic review (+/- meta-analysis) of diagnostic test accuracy (DTA) studies. | 1 |
| Abstract | 2 | Abstract: See PRISMA-DTA for abstracts. | 1-2 |
| INTRODUCTION | | | |
| Rationale | 3 | Describe the rationale for the review in the context of what is already known. | 4 |
| Clinical role of index test | D1 | State the scientific and clinical background, including the intended use and clinical role of the index test, and if applicable, the rationale for minimally acceptable test accuracy (or minimum difference in accuracy for comparative design). | 4 |
| Objectives | 4 | Provide an explicit statement of question(s) being addressed in terms of participants, index test(s), and target condition(s). | 5 |
| METHODS | | | |
| Protocol and registration | 5 | Indicate if a review protocol exists, if and where it can be accessed (e.g., Web address), and, if available, provide registration information including registration number. | 5 |
| Eligibility criteria | 6 | Specify study characteristics (participants, setting, index test(s), reference standard(s), target condition(s), and study design) and report characteristics (e.g., years considered, language, publication status) used as criteria for eligibility, giving rationale. | 5-6 |
| Information sources | 7 | Describe all information sources (e.g., databases with dates of coverage, contact with study authors to identify additional studies) in the search and date last searched. | 5-6 |
| Search | 8 | Present full search strategies for all electronic databases and other sources searched, including any limits used, such that they could be repeated. | 5-6 |
| Study selection | 9 | State the process for selecting studies (i.e., screening, eligibility, included in systematic review, and, if applicable, included in the meta-analysis). | 5-6 |
| Data collection process | 10 | Describe method of data extraction from reports (e.g., piloted forms, independently, in duplicate) and any processes for obtaining and confirming data from investigators. | 6-7 |
| Definitions for data extraction | 11 | Provide definitions used in data extraction and classifications of target condition(s), index test(s), reference standard(s) and other characteristics (e.g. study design, clinical setting). | 6-7 |
| Risk of bias and applicability | 12 | Describe methods used for assessing risk of bias in individual studies and concerns regarding the applicability to the review question. | 7-8 |
| Diagnostic accuracy measures | 13 | State the principal diagnostic accuracy measure(s) reported (e.g. sensitivity, specificity) and state the unit of assessment (e.g. per-patient, per-lesion). | 8-9 |
| Synthesis of results | 14 | Describe methods of handling data, combining results of studies and describing variability between studies. This could include, but is not limited to: a) handling of multiple definitions of target condition. b) handling of multiple thresholds of test positivity, c) handling multiple index test readers, d) handling of indeterminate test results, e) grouping and comparing tests, f) handling of different reference standards | 8-9 |
| Meta-analysis | D2 | Report the statistical methods used for meta-analyses, if performed. | 8-9 |
| Additional analyses | 16 | Describe methods of additional analyses (e.g., sensitivity or subgroup analyses, meta-regression), if done, indicating which were pre-specified. | 8-9 |
| RESULTS | | | |
| Study selection | 17 | Provide numbers of studies screened, assessed for eligibility, included in the review (and included in meta-analysis, if applicable) with reasons for exclusions at each stage, ideally with a flow diagram | 9-10 |
| Study characteristics | 18 | For each included study provide citations and present key characteristics including: a) participant characteristics (presentation, prior testing), b) clinical setting, c) study design, d) target condition definition, e) index test, f) reference standard, g) sample size, h) funding sources | 10-11 |
| Risk of bias and applicability | 19 | Present evaluation of risk of bias and concerns regarding applicability for each study. | 12 |
| Results of individual studies | 20 | For each analysis in each study (e.g. unique combination of index test, reference standard, and positivity threshold) report 2x2 data (TP, FP, FN, TN) with estimates of diagnostic accuracy and confidence intervals, ideally with a forest or receiver operator characteristic (ROC) plot. | NA |
| Synthesis of results | 21 | Describe test accuracy, including variability; if meta-analysis was done, include results and confidence intervals | 12-16 |
| Additional analysis | 23 | Give results of additional analyses, if done (e.g., sensitivity or subgroup analyses, meta-regression; analysis of index test: failure rates, proportion of inconclusive results, adverse events). | 12-16 |
| DISCUSSION | | | |
| Summary of evidence | 24 | Summarize the main findings including the strength of evidence | 16-19 |
| Limitations | 25 | Discuss limitations from included studies (e.g. risk of bias and concerns regarding applicability) and from the review process (e.g. incomplete retrieval of identified research). | 16-19 |
| Conclusions | 26 | Provide a general interpretation of the results in the context of other evidence. Discuss implications for future research and clinical practice (e.g. the intended use and clinical role of the index test) | 20 |
| OTHER | | | |
| Funding | 27 | For the systematic review, describe the sources of funding and other support and the role of the funders | 20 |

Adapted From: McInnes MDF, Moher D, et al. The PRISMA-DTA Group (2018). Preferred Reporting Items for a Systematic Review and Meta-analysis of Diagnostic Test Accuracy Studies: The PRISMA-DTA Statement. JAMA. 2018 Jan 23;319(4):388-396. doi: 10.1001/jama.2017.19163.

**Table S3. Keywords and search results in different databases**

| **Database** | **Keyword** | **Date** | **Results** |
| --- | --- | --- | --- |
| Pubmed | ((Brain tumor OR Brain neoplasms OR Gliomas OR Meningioma OR Pituitary Neoplasms OR Brain Stem Neoplasm OR brain metastasis) AND (MRI imaging OR magnetic resonance imaging) AND (deep learning OR convolutional network OR machine learning OR artificial intelligence)  AND (identification OR diagnosis OR segmentation OR detection OR identification OR sensitivity OR specificity OR ROC OR AUC)) | 2023/7/25 | 1808 |
| Embase | ((Brain tumor OR Brain neoplasms OR Gliomas OR Meningioma OR Pituitary Neoplasms OR Brain Stem Neoplasm OR brain metastasis) AND (MRI imaging OR magnetic resonance imaging) AND (deep learning OR convolutional network OR machine learning OR artificial intelligence)  AND (identification OR diagnosis OR segmentation OR detection OR identification OR sensitivity OR specificity OR ROC OR AUC)) | 2023/7/25 | 3705 |
| Web of Science | ((Brain tumor OR Brain neoplasms OR Gliomas OR Meningioma OR Pituitary Neoplasms OR Brain Stem Neoplasm OR brain metastasis) AND (MRI imaging OR magnetic resonance imaging) AND (deep learning OR convolutional network OR machine learning OR artificial intelligence)  AND (identification OR diagnosis OR segmentation OR detection OR identification OR sensitivity OR specificity OR ROC OR AUC)) | 2023/7/25 | 2203 |
| Cochrane library | ((Brain tumor OR Brain neoplasms OR Gliomas OR Meningioma OR Pituitary Neoplasms OR Brain Stem Neoplasm OR brain metastasis) AND (MRI imaging OR magnetic resonance imaging) AND (deep learning OR convolutional network OR machine learning OR artificial intelligence)  AND (identification OR diagnosis OR segmentation OR detection OR identification OR sensitivity OR specificity OR ROC OR AUC)) | 2023/7/25 | 45 |
| IEEE | ((Brain tumor OR Brain neoplasms OR Gliomas OR Meningioma OR Pituitary Neoplasms OR Brain Stem Neoplasm OR brain metastasis) AND (MRI imaging OR magnetic resonance imaging) AND (deep learning OR convolutional network OR machine learning OR artificial intelligence)  AND (identification OR diagnosis OR segmentation OR detection OR identification OR sensitivity OR specificity OR ROC OR AUC)) | 2023/7/25 | 1081 |

**Table S4: Study details, data selection criteria and indication quality**

| **Study** | **Data selection criteria** | | **Dataset specification** | | | **Indicator definition** | | |
| --- | --- | --- | --- | --- | --- | --- | --- | --- |
|  | **Inclusion criteria** | **Exclusion criteria** | **Data set** | **Data Source** | **Open data** | **Indicator standard** | **Annotation Reference** | **Exclusion poor quality** |
| Qu et al. (2023) [27] | Initially detected BMs were confirmed by pathology, or with a defined history of primary malignant tumors and confirmed by follow-up MR indications | Evidence of other intracranial neoplasms or meningeal metastasis, history of brain surgery or radiotherapy. | I Local dataset (N=1000);  G Local dataset (N=100);  T1 Local dataset (N=428);  T2 Local dataset (N=64) | West China Tianfu Hospital; Chengdu Shang Jin Nan Fu Hospital; Sichuan Cancer Hospital; West China Hospital. | NR | Two neuroradiologists reviewed the images and referred to the patients’ clinical histories, radiology reports, follow-up MRIs, and pathology results if available | Manual | Yes |
| Yin et al. (2022) [28] | Newly diagnosed with BMs, with extracranial primary tumor(s) confirmed by pathology, only the initial scan with at least one lesion was assessed | With primary intracranial tumor, with meningeal metastasis, who had undergone brain surgery, with excessive artifacts | I Local dataset (N=850);  T Local dataset (N=90);  G1 Local dataset (N=70);  G2 Local dataset (N=70);  G3 Local dataset (N=70) | Sun Yat-sen University Cancer Center, Meizhou People’s Hospital, Dongguan People’s Hospital, and Fujian Cancer Hospital | NR | Manually established by marking a rectangular box around each BM, was done by four radiologists, confirmed by two neuroradiologists | Manual | Yes |
| Pflüger et al. (2022) [29] | Adult patients with BM from several primary cancers, who underwent standardized MRI examination for radiation treatment planning | No | I Local dataset (N=308);  G Local dataset (N=30) | University of Heidelberg | NR | Ground-truth segmentation an in-training radiologist and subsequently checked by a board-certified neuroradiologist | Manual | NR |
| Ottesen et al. (2022) [30] | Patients eligible for stereotactic radiotherapy | NR | I Stanford dataset (N=156); G OUH dataset (N=65); | Oslo University Hospital Stanford University | Stanford | Established by two working radiologists | Manual | NR |
| Liang et al. (2022) [31] | Patients with metastatic brain tumors | Patients with incomplete sequences, slice thickness of >5.5 mm, significant imaging artifacts, errors in the image standardization | I Local dataset (N=326);  G Local dataset (N=81) | NRG Oncology CC001 phase 3 trial | NR | Manually delineated by 2 investigators and reviewed by a senior central nervous system radiation oncologist | Manual | Yes |
| Kang et al. (2022) [32] | Radiologically diagnosed meningioma by MRI | Under 18 years old, Multiple meningiomas, Orbital meningioma, Any prior treatment for intracranial meningioma before registration | I Local dataset (N=559);  G Local dataset (N=100) | Seoul National University Hospital | NR | Two neurosurgeons manually segmented the tumor on the software and cross-checked each other's segmentation result for use as the ground truth | Manual | NR |
| Chakrabarty et al. (2022 [33]) | Pathologically confirmed glioma (CNS WHO grades 2‐4) from preoperative patients with no prior resection | NR | I BRATS2021(N=1251);  G1 Local dataset (N=384); G2 Local dataset (N=30) | Washington University School of Medicine, University of Texas MD Anderson Cancer Center | BRATS | Independently reviewed by three clinical experts; refined by a single clinical expert with clinical oversight from a second expert | Manual | NR |
| Abayazeed et al. (2022) [34] | Patients who glioma | NR | I1 TCIA dataset (300);  I2 Local dataset (N=46);  G Local dataset (N=20) | The Cancer Imaging Archive; University of Florida, Gainesville FL, Houston Methodist Hospital, Houston TX and JFK Medical Center; University of California, Los Angeles.; | TCIA | Six neuroradiologists each with *>*6 years of neuroradiology practice experience created the ground truth (GT) with 2 rounds of overreads for consensus; Two neuroradiologists from UCLA created the GT for the External validation dataset using the institution standards of practice and no over-read. | Manual | NR |
| Ma et al. (2022) [35] | Meningioma patients | NR | I Local dataset (N=501);  G Local dataset (N=50) | Huashan Hospital, Gamma Knife Hospital, Huashan Hospital North | NR | Ground truth manually annotated by radiologists | Manual | NR |
| Chen et al. (2022) [36] | Patients underwent complete MRI examination before treatment, patients had high-quality MRI data, available clinicopathological data, only a single meningioma was detected in each patient | Patients with history of brain tumor | I Local dataset (N=545);  G Local dataset (N=64) | Sun Yat-Sen University Cancer Centre, Xiangya Hospital Central South University | NR | Delineated jointly by two readers and reviewed by another senior reader | Manual | Yes |
| Yi et al. (2021) [37] | Receive stereotactic radiosurgery for at least one brain metastasis measured to a minimum of 5 mm in one direction, be untreated or progressive after systemic or local therapy, have confirmed non-small-cell lung cancer or malignant melanoma, be ≥18 years of age; have an Eastern Cooperative Oncology Group performance status score of maximum 1, and have a life expectancy of more than 6 weeks | NR | I Stanford dataset (N=156); G OUH dataset (N=65) | Stanford, Oslo University Hospital | Stanford | NR | Manual | NR |
| Rudie et al. (2021) [38] | Undergoing stereotactic radiosurgery planning | Patients without definitive enhancing intracranial metastases, Patients who presented with only dura-based or leptomeningeal metastases, examinations that had missing sequences or yielded corrupted data, patients with resection cavities | I Local dataset (N=313);  G Local dataset (N=100) | University of California | NR | Hand-segmented by a neuroradiology fellow or attending neuroradiologist with the final radiology report used as a reference | Manual | Yes |
| Cho et al. (2021) [39] | Patients who had a confirmed systemic malignancy and underwent MRI using a “BM work-up” protocol | Patients with a history of primary brain cancers, presence of metastasis involving the bone, dura, or skin or suspicious lesions for leptomeningeal seeding, presence of other pathological conditions, presence of equivocal nodule(s) determined to be BM, presence of excessive artifacts or poor image quality, presence of more than 50 metastatic nodules, absence of BM | I T Local dataset (N=159); G Local dataset (N=35) | Seoul National University Bundang Hospital, Seoul National University Hospital | NR | Two radiologists reviewed the data. All the radiologists had access to the patients’ histories and follow-up imaging studies and determined the reference standard of BM nodules based on consensus. | Manual | Yes |
| Laukamp et al. (2021) [40] | Consecutive patients with intracranial meningiomas and available MRI data were referred to our institution for surgical treatment | Incomplete multiparametric MRI dataset (T1-weighted and T2-weighted, FLAIR and T1CE), missing histological specimens, previous treatment, recurrent or multiple meningiomas and/or a second prevalent tumor entity; severe leukoencephalopathy potentially impairing FLAIR tumor segmentation; severe MRI artifacts | I Local dataset (N=70);  T Local dataset (N=56) | University Hospital Cologne | NR | Performed independently by two radiologists; | Manual | Yes |
| Takahashi et al. (2021) [41] | Surgical removal or biopsy was performed. Diagnostic tests, including genetic analysis of key biomarkers (IDH mutation and 1p19q), were performed following the WHO 2007 or 2016 classifications of Central Nerves System tumors. | Missing MRI sequences | I BRATS2019(N=235);  G Local dataset (N=544) | 10 facilities in Japan | BRATS | Manually created by skilled neuroradiologists using in-house tools | Manual | NR |
| Grøvik et al. (2021) [42] | Presence of known or possible metastatic disease, and the availability of all required MR imaging sequences. Only patients with ≥1; measured at a minimum of 5 mm in one direction, be untreated or progressive after systemic or local therapy, have confirmed non-small-cell lung cancer (NSCLC) or malignant melanoma, be ≥18 years of age; have an Eastern Cooperative Oncology Group performance status score ≤1, and have a life expectancy >6 week metastatic lesion were included | Prior surgical or radiation therapy | I Local dataset (N=100);  G Local dataset (N=65) | Oslo University Hospital and Stanford Review Board | NR | Neuroradiologist with 3 years of experience manually delineated each enhancing brain lesion by placing a region of interest (ROI) over each image slice | Manual | NR |
| Conte et al. (2021) [43] | Histologic diagnosis of glioblastoma (GBM) or central nervous system lymphoma (CNSL) and availability of precontrast T1-weighted, T2-weighted, and FLAIR MRI scans and postcontrast T1-weighted MRI scans | NR | I BRATS2017(N=210);  G Local dataset (N=67) | University of Pavia, | BRATS | NR | NR | NR |
| Bouget et al. (2021) [44] | Patients of at least 18 years old with a newly diagnosed glioblastoma at first-time surgery | NR | I Local dataset (N=1534);  G BRATS2020(N=293) | **ALK, AMS, GRO, HAG, MIL, PAR, SFR, SLO, TIL, UTR, VIE, ZWO, STO | BRATS | Manually segmented in 3D by trained raters using an initiation by either a region growing algorithm or a grow cut algorithm and subsequent manual editing. Trained raters were supervised by neuroradiologists and neurosurgeons | Manual | NR |
| Sunwoo et al. (2017) [45] | Consecutive MR imaging studies collected using a ‘BM work-up’ protocol from patients who had confirmed systemic malignancy | Presence of metastasis involving bone, dura, or skin, or suspicious lesions for leptomeningeal seeding; Presence of other pathological conditions, such as meningioma, vestibular schwannoma, pituitary adenoma, cavernous malformation, or hemorrhagic infarction; Presence of equivocal nodule(s) determined to be BM; Presence of excessive artifacts or poor image quality; Presence of more than 50 metastatic nodule | I Local dataset (N=798);  T Local dataset (N=266) | Seoul National University Bundang Hospital | NR | NR | NR | Yes |

Abbreviation: NR: not recorded; BM, brain metastasis, MR, Magnetic resonance imaging; MRI, Magnetic resonance imaging; CE, contrast enhanced; I, internal validation; T, temporal validation; G, geographical validation;

**ALK, Northwest Clinics, Alkmaar, Netherlands; AMS, Amsterdam University Medical Centers, location VU medical center, Netherlands; GRO, University Medical Center Groningen, Netherlands; HAG, Medical Center Haaglanden, the Hague, Netherlands; MIL, Humanitas Research Hospital, Milano, Italy; PAR, Hôpital Lariboisière, Paris, France; SFR, University of California San Francisco Medical Center, U.S.; SLO, Medical Center Slotervaart, Amsterdam, Netherlands; TIL, St Elisabeth Hospital, Tilburg, Netherlands; UTR, University Medical Center Utrecht, Netherlands; VIE, Medical University Vienna, Austria; ZWO, Isala hospital, Zwolle, Netherlands; STO, the St. Olavs hospital, Trondheim University Hospital, Norway (STO)

**Table S5: MRI infrastructure details**

| **Study** | **Scanning Protocols** | | **Scanner specification** | | | |
| --- | --- | --- | --- | --- | --- | --- |
|  | **Slice Thickness** | **Scanning Sequences** | **Tesla** | **Manufacturer** | **Model** | **Scanner** |
| Qu et al. (2023) [28] | 1 mm | 3D T1c | 1.5T/3T | 4 | 9 | United Imaging-uMR 588; United Imaging-uMR 780; Siemens-Avanto; Siemens-Trio Tim; Siemens-Skyra; GE-SIGNA Architect; GE-Discovery; GE-SIGNA Primier; Philips-Ingenia Elition |
| Yin et al. (2022) [29] | 1 mm | 3D T1c | 1.5T/3T | 4 | 13 | Training: Siemens MAGNETOM Aera; Philips Achieva ; GE Discovery MR750; GE Discovery MR750W; GE SIGNA Pioneer; GE SIGNA Premier; GE SIGNA Architect; Philips Ingenia CX; Siemens MAGNETOM Trio Tim; Siemens MAGNETOM Prisma; United Imaging uMR560; United Imaging uMR780; United Imaging uMR790;  Validation: GE Optima MR360; Siemens MAGNETOM Skyra; Siemens MAGNETOM Prisma, Siemens MAGNETOM Verio; GE Discovery MR750w |
| Pflüger et al. (2022) [30] | 1mm; 5mm | 3D T1c, 3D T1, 2D T1c, 2D T1, FLAIR | 1.5T/3T | 1 | 4 | Training: SIEMENS Magnetom Verio; SIEMENS Skyra; SIEMENS Trio TIM; SIEMENS Magnetom Avanto;  Validation: SIEMENS Magnetom Avanto |
| Ottesen et al. (2022) [31] | 0.9 m; 1 to 1.6 mm | 3D T1c, 3D T1, 3D T1 BRAVO, 3D FLAIR | 1.5T/3T | 2 | 6 | Training: GE SIGNA Explorer; GE TwinSpeed; GE Discovery 750; GE 750w; GE SIGNA Architect; Simen Skyra |
| Liang et al. (2022) [32] | 0.43 mm to 7.22 mm | T1c, T2 FLAIR | NR | 4 | 14 | NR (The MR images were acquired on 14 types of scanners from 4 major vendors—Siemens, GE, Philips, and Toshiba). |
| Kang et al. (2022) [33] | 6mm to 7mm (median) | 3D T1c | 1T/1.5T/3T | 6 | 31 | Training: GE Discovery MR750w; GE Genesis Signa; GE Signa Excite; GE Signa HDe; GE Signa HDx; GE Signa HDxt; HITACHI AIRIS II; ISOL Technology Chorus; MEDINUS Magnum; PHILIPS Achieva; Philips Achieva dStream; Philips Gyroscan Intera; Philips Gyroscan NT; Philips Ingenia; Philips Ingenia CX; Philips Intera; SIEMENS Avanto; SIMENS Espree; SIMENS Expert; SIMENS Harmony; SIMENS Romio; SIMENS Skyra; SIMENS Skyra Fit; SIMENS Sonata; SIMENS Trio; SIMENS Trio Tim; SIMENS Symphony; SIMENS Symphony Tim; SIMENS Verio; SIMENS Vision; SIMENS Vision Plus  Internal Validation: GE Discovery MR750w; GE Genesis Signa; GE Signa Excite; GE Signa HDx; GE Signa HDxt; MEDINUS Magnum; PHILIPS Achieva; Philips Achieva dStream; Philips Ingenia; Philips Ingenia CX; Philips Intera; SIEMENS Avanto; SIMENS Expert; SIMENS Harmony; SIMENS Romio; SIMENS Skyra; SIMENS Skyra Fit; SIMENS Sonata; SIMENS Trio Tim; SIMENS Verio; SIMENS Vision Plus  Geographical Validation: GE Discovery MR750w; GE Genesis Signa; GE Optima 360; GE Signa Excite; GE Signa HDe; GE Signa HDxt; HITACHI Aperto Eterna; PHILIPS Achieva; Philips Gyroscan Intera; Philips Gyroscan NT; Philips Ingenia; Philips Intera; SIEMENS Avanto; SIMENS C!; SIMENS Expert; SIMENS Skyra; SIMENS Sonata; SIMENS Trio Tim; SIMENS Symphony; SIMENS SIMENS Verio; SIMENS Vision; TOSHIBA MRT200PP3 |
| Chakrabarty et al. (2022 [34]) | NR | 3D T1c, T1, 2D T2, 2D FLAIR | NR | NR | NR | NR |
| Abayazeed et al. (2022) [35] | 5 mm | T1c, T1, T2, T2 FLAIR | NR | NR | NR | NR |
| Ma et al. (2022) [36] | 2mm; 3mm | T1c | 1.5T/3T | 2 | NR | GE Milwaukee, Siemens Healthcare |
| Chen et al. (2022) [37] | 5mm to 6mm | T1c, T1, T2 | NR | 3 | 9 | Training: Philips Achieva; GE DISCOVERY MR750; GE DISCOVERY MR750w; GE SIGNA EXCITE; GE SIGNA; GE Signa HDxt; SIEMENS Espree; SIEMENS TrioTim  Temporal Validation: Philips Achieva, Philips Panorama HFO; GE DISCOVERY MR750, GE DISCOVERY MR750w ; GE SIGNA EXCITE; GE Signa HDxt; GE SIGNA Pioneer; SIEMENS Aera, SIEMENS Espree; SIEMENS TrioTim; United Imaging uMR  Geographical Validation: Alltech Centauri, GE Signa HDxt, SIEMENS Aera, SIEMENS Sonata; TOSHIBA_MEC MRT200SP3 |
| Yi et al. (2021) [38] | NA | 3D T1c, 3D T1, 3D T1 BRAVO, 3D FLAIR | 1.5T/3T | 2 | 6 | GE SIGNA Explorer and TwinSpeed ; GE Discovery 750; GE 750 w and SIGNA Architect; Skyra, Siemens Healthineers |
| Rudie et al. (2021) [39] | $\leq$1.50 mm | T1c, T1 | 1.5T/3T | 2 | 3 | GE Signa HDxt; Philips Achieva; GE Discovery MR750 |
| Cho et al. (2021) [40] | 1 mm | 3D T1c | 1.5T/3T | 1 | 3 | Training: Philips Intera; Philips Achieva; Philips Ingenia;  Validation: SIEMENS Verio; GE Discovery 750w |
| Laukamp et al. (2021) [41] | 3.75mm to 6.09mm (mean) | T1c, T2 FLAIR | 1T/1.5T/3T | 4 | 13 | Training: Philips Achieva; Philips Ingenia; Philips Intera; Philips Panorama; Siemens Aera; Siemens Avanto; Siemens Espree; Siemens Prisma; Siemens Symphony; Siemens Verio; Toshiba Titan; Toshiba Vantage Elan; General Electric Optima  Validation: Philips Achieva; Philips Gyroscan; Philips Ingenia; Philips Intera; Philips Panorama; Siemens Aera; Siemens Avanto; Siemens Espree; Siemens Symphony; Siemens Verio; Toshiba Titan; |
| Takahashi et al. (2021) [42] | 3.3 mm to 9mm | T1c, T1, T2, T2 FLAIR | NR | NR | NR | NR |
| Grøvik et al. (2021) [43] | 1mm to 1.6mm | 3D T1c, 3D T1, 3D T1 BRAVO, 3D FLAIR | 1.5T/3T | 2 | 6 | Training: GE TwinSpeed; GE SIGNA Explorer; GE SIGNA Architect; GE Discovery 750; GE 750w; Siemens Skyra  Validation: Siemens Skyra |
| Conte et al. (2021) [44] | NR | T1c, T1, T2, T2 FLAIR | NR | NR | NR | NR |
| Bouget et al. (2021) [45] | 1mm | T1c | 1.5T/3T | 3 | 10 | Siemens model Sonata; Siemens Avanto; Siemens Skyra; Siemens Prisma; Siemens mMR; GE model Signa HDxt; GE DISCOVERY MR750; GE Toshiba; GE model Titan3T; Philips model Panorama HFO; Philips Ingenuity |
| Sunwoo et al. (2017) [46] | 1mm | 3D T1c | 1.5T/3T | 1 | 2 | Philips Intera; Philips Achieva |

Abbreviation: NR: not recorded; GE, General Electric healthcare

**Table S6: Technical detailed of model constriction**

| **Study** | **Preprocessing** | | | | | | | | **Model training** | | | |
| --- | --- | --- | --- | --- | --- | --- | --- | --- | --- | --- | --- | --- |
|  | **N4 bias correction** | **Pixel resampling** | **Skull stripping** | **Data augmentation** | **Normalization** | **Other preprocessing** | **Cropping image** | **Transfer learning** | | **Loss function** | **Two-stage** | **Ensemble** |
| Qu et al. (2023) [27] | No | No | Yes | No | Yes | Gamma correction | No | No | | Tversky loss | No | No |
| Yin et al. (2022) [28] | No | No | No | Yes | No | NR | Yes | No | | Focal loss | Yes | No |
| Pflüger et al. (2022) [29] | No | Yes | Yes | Yes | Yes | NR | Yes | No | | Soft dice loss and cross entropy loss | No | Yes |
| Ottesen et al. (2022) [30] | No | Yes | Yes | Yes | Yes | Input-level dropout | Yes | No | | Focal Tversky and binary cross entropy | No | No |
| Liang et al. (2022) [31] | Yes | Yes | Yes | Yes | Yes | NR | Yes | No | | Dice loss | No | No |
| Kang et al. (2022) [32] | No | Yes | No | Yes | Yes | NR | Yes | No | | Dice loss and cross entropy loss | No | Yes |
| Chakrabarty et al. (2022 [33]) | No | Yes | Yes | Yes | Yes | NR | No | No | | Multi-class dice loss function | No | No |
| Abayazeed et al. (2022) [34] | No | No | Yes | Yes | Yes | NR | Yes | No | | NR | No | Yes |
| Ma et al. (2022) [35] | No | No | No | No | Yes | Histogram equalization | Yes | No | | Binary focal loss and the Dice loss | No | No |
| Chen et al. (2022) [36] | Yes | No | No | Yes | Yes | NR | Yes | No | | Dice loss and cross entropy loss | No | No |
| Yi et al. (2021) [37] | No | No | No | No | No | Histogram equalization; Input-level dropout | No | No | | Cross entropy loss | No | Np |
| Rudie et al. (2021) [38] | No | Yes | No | Yes | Yes | NR | Yes | No | | Soft dice loss, cross entropy loss, balanced cross entropy loss and focal loss | No | No |
| Cho et al. (2021) [39] | No | Yes | No | Yes | Yes | NR | No | No | | Dice loss | Yes | No |
| Laukamp et al. (2021) [40] | Yes | Yes | Yes | Yes | Yes | NR | Yes | No | | Cross entropy loss | No | No |
| Takahashi et al. (2021) [41] | No | Yes | Yes | No | No | NR | Yes | No | | NR | No | No |
| Grøvik et al. (2021) [42] | No | No | No | No | No | Input-level dropout | No | No | | NR | No | No |
| Conte et al. (2021) [43] | No | Yes | Yes | Yes | Yes | GAN missing sequence | Yes | No | | Soft dice loss | No | No |
| Bouget et al. (2021) [44] | No | Yes | Yes | Yes | Yes | NR | No | No | | Class-averaged Dice loss | No | No |
| Sunwoo et al. (2017) [45] | No | No | Yes | No | Yes | NR | No | No | | NR | No | No |

Abbreviation: NR: not recorded; GAN: generative adversarial network

**Table S7: Algorithms related technical factors as moderator or subgroup to algorithms segmentation performance**

|  | Model input | | | Algorithms | Preprocessing | | | | | |
| --- | --- | --- | --- | --- | --- | --- | --- | --- | --- | --- |
|  | Training size | Model input sequence | Model input dimension | Algorithms types | N4 bias field correction | Pixel resampling | Skull stripping | Data augmentation | Intensity normalization | Cropping image |
| External validation set, best accuracy (k=15) | Coeff = 0.0000667;  p=0.187 | Q=1.34;  p=0.25 | Q=16.19;  p<0.01* | Q=47.34;  p<0.01* | Q=16.74;  p<0.01* | Q=4.59;  p=0.03* | Q=0.77;  p=0.38 | Q=0.42;  p=0.52 | Q=2.64;  p=0.10 | Q=2.61;  p=0.11 |
| All validation set, best accuracy algorithms (k=20) | Coeff = 0.0000696;  p=0.090 | Q=0.92;  p=0.34 | Q=13.20;  p<0.01* | Q=30.57;  p<0.01* | Q=13.80;  p<0.01* | Q=1.83;  p=0.18 | Q=1.00;  p=0.32 | Q=0.54;  p=0.46 | Q=4.34;  p=0.04* | Q=1.12;  p=0.29 |
| All validation set, all algorithms (k=58) | Coeff = 0.0000575;  p=0.109 | Q=3.21;  p=0.07 | Q=17.20;  p<0.01* | Q=13.22;  p<0.01* | Q=37.10;  p<0.01* | Q=1.97;  p=0.16 | Q=3.33;  p=0.07 | Q=11.87;  p<0.01* | Q=6.52;  p=0.01* | Q=0.13;  p=0.72 |
| All validation set, all algorithms (three-level) (k=58) | p=0.295 | p=0.196 | p=0.013* | p<0.01* | p=0.035* | p=0.708 | p=0.268 | p<0.009* | p=0.185 | p=0.357 |

**Table S8: Algorithms related technical factors as moderator or subgroup to algorithms detection performance**

|  | Model input | | | | Algorithms | Preprocessing | | | | | |
| --- | --- | --- | --- | --- | --- | --- | --- | --- | --- | --- | --- |
|  | Training size | Lesion number | Model input sequence | Model input dimension | Algorithms types | N4 bias field correction | Pixel resampling | Skull stripping | Data augmentation | Intensity normalization | Cropping image |
| Patient-wise sensitivity | | | | | | | | | | | |
| External validation set, best accuracy (k=13) | Coeff=-6.73e-06  p=0.961 | Coeff= 9.51e-06  p=0.202 | Q=0.04;  p=0.83 | Q=0.03;  p=0.87 | Q=0.54;  P=0.77 | Q=1.78;  p=0.18 | Q=3.31;  p=0.07 | Q=0.39;  p=0.53 | Q=0.06;  p=0.81 | Q=8.83;  p<0.01* | Q=2.36;  p=0.12 |
| All validation set, best accuracy algorithms (k=17) | Coeff= 7.82e-7  p=0.994 | Coeff= 8.86e-6  p=0.122 | Q=1.13;  p=0.72 | Q=1.98;  p=0.37 | Q=26.37;  p<0.01* | Q=0.62;  p=0.43 | Q=4.01;  p=0.05 | Q=0.12;  p=0.73 | Q=0.02;  p=0.89 | Q=12.12;  p<0.01* | Q=3.36;  p=0.07 |
| All validation set, all algorithms (k=27) | Coeff= .0000412  p=0.628 | Coeff= 7.79e-06  p=0.056 | Q=0.03;  p=0.87 | Q=3.72;  p=0.16 | Q=12.99;  p=0.01* | Q=1.21;  p=0.27 | Q=3.21;  p=0.07 | Q=0.00;  p=0.94 | Q=0.36;  p=0.55 | Q=22.26;  p<0.01* | Q=3.60;  p=0.06 |
| Lesion-wise sensitivity | | | | | | | | | | | |
| External validation set, best accuracy (k=13) | Coeff= 0.000053  p=0.731 | Coeff= 0.000013  p=0.089 | Q=0.07;  p=0.79 | Q=7.79;  p=0.22 | Q=6.17  P=0.10 | Q=0.56;  p=0.76 | Q=3.60;  p=0.06 | Q=0.72;  p=0.02* | Q=0.24;  p=0.62 | Q=11.26  p<0.01* | Q=2.26;  p=0.11 |
| All validation set, best accuracy algorithms (k=17) | Coeff= 0.0001  p=0.303 | Coeff= 0.000010  p=0.025* | Q=0.18;  p=0.67 | Q=3.84;  p=0.15 | Q=24.00  p<0.01* | Q=0.86;  p=0.35 | Q=6.38;  p=0.01* | Q=0.15;  p=0.70 | Q=0.06;  p=0.81 | Q=12.54  p<0.01* | Q=3.24;  p=0.07 |
| All validation set, all algorithms (k=27) | Coeff= 0.000096  p=0.365 | Coeff= 0.000011  p=0.031* | Q=0.03;  p=0.86 | Q=7.79;  p=0.02* | Q=14.29  p=0.01* | Q=1.04;  p=0.31 | Q=5.3;  p=0.02* | Q=0.01;  p=0.90 | Q=0.05;  p=0.82 | Q=21.55  p<0.01* | Q=3.69;  p=0.05 |

**Table S9. Quality assessment according to the Quality Assessment of Diagnostic Accuracy Studies 2 (QUADAS-2) criteria**

| Source | Risk of bias | | | | | | | | | | | Concern of applicability | | |
| --- | --- | --- | --- | --- | --- | --- | --- | --- | --- | --- | --- | --- | --- | --- |
|  | Patient selection: | | | INDEX TEST | | Reference Standard | | Flow and Timing | | | | Patient selection | INDEX TEST | Reference Standard |
|  | Consecutive | Case- control | Inappropriate exclusions | Blind to reference standard | Threshold prespecified | Correctly classify the target condition | Blind to index test | Appropriate interval | Receive a reference standard | Same reference standard | All patients analyzed |  |  |  |
| Qu et al. (2023) [27] | Yes | No | No | Yes | Yes | Yes | Yes | Yes | Yes | Yes | Yes | Low | Low | Low |
| Yin et al. (2022) [28] | Yes | No | No | Yes | Yes | Yes | Yes | Yes | Yes | Yes | Yes | Low | Low | Low |
| Pflüger et al. (2022) [29] | Yes | No | No | Yes | Yes | Yes | Yes | Yes | Yes | Yes | Yes | Low | Low | Low |
| Ottesen et al. (2022) [30] | Unclear | No | No | Yes | Yes | Yes | Yes | Yes | Yes | Yes | Yes | Low | Low | Low |
| Liang et al. (2022) [31] | Yes | No | No | Yes | Yes | Yes | Yes | Yes | Yes | Yes | Yes | Low | Low | Low |
| Kang et al. (2022) [32] | Yes | No | No | Yes | Yes | Yes | Yes | Yes | Yes | Yes | Yes | Low | Low | Low |
| Chakrabarty et al. (2022 [33] | Unclear | No | No | Yes | Yes | Yes | Yes | Yes | Yes | Yes | Yes | Low | Low | Low |
| Abayazeed et al. (2022) [34] | Unclear | No | No | Yes | Yes | Yes | Yes | Yes | Yes | Yes | Yes | Low | Low | Low |
| Ma et al. (2022) [35] | Unclear | No | No | Yes | Yes | Yes | Yes | Yes | Yes | Yes | Yes | Low | Low | Low |
| Chen et al. (2022) [36] | Yes | No | No | Yes | Yes | Yes | Yes | Yes | Yes | Yes | Yes | Low | Low | Low |
| Yi et al. (2021) [37] | Unclear | No | Yes | Yes | Yes | Yes | Yes | Yes | Yes | Yes | Yes | Low | Low | Low |
| Rudie et al. (2021) [38] | Unclear | No | No | Yes | Yes | Yes | Yes | Yes | Yes | Yes | Yes | Low | Low | Low |
| Cho et al. (2021) [39] | Yes | No | No | Yes | Yes | Yes | Yes | Yes | Yes | Yes | Yes | Low | Low | Low |
| Laukamp et al. (2021) [40] | Yes | No | No | Yes | Yes | Yes | Yes | Yes | Yes | Yes | Yes | Low | Low | Low |
| Takahashi et al. (2021) [41] | Yes | No | No | Yes | Yes | Yes | Yes | Yes | Yes | Yes | Yes | Low | Low | Low |
| Grøvik et al. (2021) [42] | Unclear | No | Yes | Yes | Yes | Yes | Yes | Yes | Yes | Yes | Yes | Low | Low | Low |
| Conte et al. (2021) [43] | Yes | No | No | Yes | Yes | Yes | Yes | Yes | Yes | Yes | Yes | Low | Low | Low |
| Bouget et al. (2021) [44] | Yes | No | No | Yes | Yes | Yes | Yes | Yes | Yes | Yes | Yes | Low | Low | Low |
| Sunwoo et al. (2017) [45] | Yes | No | No | Yes | Yes | Yes | Yes | Yes | Yes | Yes | Yes | Low | Low | Low |

**Table S10. The Checklist for Artificial Intelligence in Medical Imaging scores.**

| Source | Title/Abstract | Introduction | Methods | | | | | | | Results | | Discussion | Other Information | Total Score |
| --- | --- | --- | --- | --- | --- | --- | --- | --- | --- | --- | --- | --- | --- | --- |
|  |  |  | Study design | Data | Ground truth | Data preparation | Model | Training | Evaluation | Data | Model performance |  |  |  |
|  | (2) | (2) | (2) | (7) | (5) | (3) | (3) | (3) | (5) | (2) | (3) | (2) | (3) | (42) |
| Qu et al. (2023) [27] | 2 | 2 | 2 | 5 | 3 | 2 | 1 | 2 | 3 | 2 | 2 | 2 | 1 | 29 |
| Yin et al. (2022) [28] | 2 | 2 | 2 | 5 | 3 | 2 | 2 | 3 | 5 | 2 | 3 | 2 | 1 | 34 |
| Pflüger et al. (2022) [29] | 2 | 2 | 2 | 5 | 3 | 2 | 2 | 3 | 5 | 1 | 3 | 2 | 1 | 33 |
| Ottesen et al. (2022) [30] | 2 | 2 | 2 | 5 | 1 | 2 | 1 | 2 | 5 | 1 | 2 | 2 | 1 | 28 |
| Liang et al. (2022) [31] | 2 | 2 | 2 | 5 | 2 | 2 | 2 | 3 | 4 | 1 | 1 | 2 | 1 | 29 |
| Kang et al. (2022) [32] | 2 | 2 | 2 | 6 | 5 | 2 | 2 | 2 | 5 | 2 | 2 | 2 | 3 | 37 |
| Chakrabarty et al. (2022 [33]) | 1 | 2 | 2 | 5 | 3 | 2 | 1 | 1 | 5 | 1 | 1 | 2 | 1 | 27 |
| Abayazeed et al. (2022) [34] | 2 | 2 | 2 | 6 | 5 | 2 | 1 | 2 | 4 | 2 | 2 | 2 | 1 | 33 |
| Ma et al. (2022) [35] | 2 | 2 | 1 | 4 | 1 | 2 | 2 | 1 | 4 | 0 | 1 | 2 | 1 | 23 |
| Chen et al. (2022) [36] | 2 | 2 | 2 | 6 | 5 | 2 | 3 | 1 | 5 | 2 | 2 | 2 | 2 | 36 |
| Yi et al. (2021) [37] | 2 | 2 | 2 | 7 | 0 | 2 | 2 | 1 | 3 | 0 | 2 | 1 | 1 | 25 |
| Rudie et al. (2021) [38] | 2 | 2 | 2 | 6 | 5 | 2 | 2 | 3 | 5 | 1 | 2 | 2 | 1 | 35 |
| Cho et al. (2021) [39] | 2 | 2 | 2 | 6 | 4 | 2 | 1 | 3 | 5 | 1 | 1 | 2 | 1 | 32 |
| Laukamp et al. (2021) [40] | 2 | 2 | 2 | 6 | 4 | 2 | 1 | 3 | 5 | 1 | 1 | 2 | 1 | 32 |
| Takahashi et al. (2021) [41] | 2 | 2 | 2 | 4 | 3 | 2 | 3 | 2 | 5 | 1 | 2 | 2 | 1 | 31 |
| Grøvik et al. (2021) [42] | 2 | 2 | 2 | 7 | 4 | 2 | 2 | 1 | 5 | 1 | 2 | 2 | 1 | 33 |
| Conte et al. (2021) [43] | 2 | 2 | 2 | 6 | 3 | 2 | 2 | 2 | 5 | 2 | 2 | 2 | 2 | 34 |
| Bouget et al. (2021) [44] | 1 | 2 | 1 | 5 | 3 | 2 | 1 | 3 | 5 | 0 | 2 | 1 | 2 | 28 |
| Sunwoo et al. (2017) [45] | 1 | 2 | 2 | 6 | 5 | 2 | 1 | 0 | 5 | 2 | 3 | 2 | 1 | 32 |
